# Contribution of the Two Genes Encoding Histone Variant H3.3 to Viability and Fertility in Mice

**DOI:** 10.1371/journal.pgen.1004964

**Published:** 2015-02-12

**Authors:** Michelle C. W. Tang, Shelley A. Jacobs, Deidre M. Mattiske, Yu May Soh, Alison N. Graham, An Tran, Shu Ly Lim, Damien F. Hudson, Paul Kalitsis, Moira K. O’Bryan, Lee H. Wong, Jeffrey R. Mann

**Affiliations:** 1 Department of Zoology, The University of Melbourne, Melbourne, Victoria, Australia; 2 Genetics Theme, Murdoch Children’s Research Institute, Parkville, Victoria, Australia; 3 Department of Anatomy and Developmental Biology, Monash University, Melbourne, Victoria, Australia; 4 Department of Biochemistry and Molecular Biology, Monash University, Melbourne, Victoria, Australia; Australia National University, UNITED STATES

## Abstract

Histones package DNA and regulate epigenetic states. For the latter, probably the most important histone is H3. Mammals have three near-identical H3 isoforms: canonical H3.1 and H3.2, and the replication-independent variant H3.3. This variant can accumulate in slowly dividing somatic cells, replacing canonical H3. Some replication-independent histones, through their ability to incorporate outside S-phase, are functionally important in the very slowly dividing mammalian germ line. Much remains to be learned of H3.3 functions in germ cell development.

Histone H3.3 presents a unique genetic paradigm in that two conventional intron-containing genes encode the identical protein. Here, we present a comprehensive analysis of the developmental effects of null mutations in each of these genes. *H3f3a* mutants were viable to adulthood. Females were fertile, while males were subfertile with dysmorphic spermatozoa. *H3f3b* mutants were growth-deficient, dying at birth. *H3f3b* heterozygotes were also growth-deficient, with males being sterile because of arrest of round spermatids. This sterility was not accompanied by abnormalities in sex chromosome inactivation in meiosis I. Conditional ablation of *H3f3b* at the beginning of folliculogenesis resulted in zygote cleavage failure, establishing *H3f3b* as a maternal-effect gene, and revealing a requirement for H3.3 in the first mitosis. Simultaneous ablation of *H3f3a* and *H3f3b* in folliculogenesis resulted in early primary oocyte death, demonstrating a crucial role for H3.3 in oogenesis.

These findings reveal a heavy reliance on H3.3 for growth, gametogenesis, and fertilization, identifying developmental processes that are particularly susceptible to H3.3 deficiency. They also reveal partial redundancy in function of *H3f3a* and *H3f3b*, with the latter gene being generally the most important.

## Author Summary

Histones package DNA and regulate chromosome activity. Histone H3 is particularly important in this regard. The H3.3 isoform is unique, among H3 histones, in being able to incorporate into chromosomes independent of DNA replication. Thus, in slowly- or non-dividing somatic cells, H3.3 histone can take on the bulk of H3 functions. The developing germ line is very slowly dividing, and undergoes large-scale changes in chromosome activity. H3.3 is therefore likely to be very important in regulating these processes. Here, we have studied the effects of null mutations in each of the two genes encoding H3.3. We demonstrate that H3.3 is very important in germ cell development, regulating oogenesis, spermatogenesis, and fertilization. Also, we reveal H3.3 to be important in somatic growth. Each of the two genes is required to varying extents in regulating these processes.

## Introduction

The core histones H2A, H2B, H3 and H4, package DNA into nucleosomes and modify higher-order chromatin structure. H3 in mammals is represented by three near-identical 135 amino acid isoforms: canonical H3.1 and H3.2, and the variant H3.3. Chromatin incorporation is replication-coupled for the canonical forms, and is replication-independent for H3.3 [1–4]. H3.3 plays fundamental roles in regulating genome function and stability. It can accumulate in terminally differentiated or non-dividing cells, and can become the predominant H3 isoform [3, 5]. Through the chaperone HIRA (histone cell cycle regulation defective homolog A (*S*. *cerevisiae*)), H3.3 is deposited at active genes and regulatory regions, forming more accessible nucleosomes [3, 4, 6–8], and underlies the formation of epigenetic bivalent domains at the promoter regions of poised genes in embryonic stem (ES) cells [8, 9]. Through the chaperones ATRX (alpha thalassemia/mental retardation syndrome X-linked) and DAXX (Fas death domain-associated protein), H3.3 is deposited at repetitive regions such as telomeres and centromeres, serving genome stabilization functions [10–12]. H3.3 is required for heterochromatin formation in mammalian preimplantation-stage embryos [13, 14], and has been reported to preferentially localize to the heterochromatic XY-body in meiosis-I, where it could serve functions related to meiotic sex chromosome inactivation (MSCI) [15]. Finally, specific amino acid substitutions in H3.3 drive cancer [16, 17], underscoring the fundamental importance of H3.3 in regulating epigenetic states.

Canonical histones are encoded by numerous clustered intronless genes, while histone variants are typically encoded by one intron-containing gene. H3.3 presents a unique genetic paradigm in that the identical protein is encoded by two intron-containing unlinked genes, *H3f3a*, and *H3f3b* (histone 3, family 3A and -B). We will refer to the encoded proteins as H3.3^A^ and H3.3^B^, respectively. The two genes have divergent regulatory and intervening regions, suggesting some qualitatively different functional roles. The presence of two H3.3-encoding genes, and their genomic arrangement, is highly conserved in mammals and birds [18].

Here, we have investigated the developmental effects of null mutations of *H3f3a* and *H3f3b* in the mouse (*Mus musculus*), each mutated by the same experimental strategy, and in the identical mouse strain—129S1/SvImJ (129S1). *H3f3a* mutants were viable to adulthood. By contrast, *H3f3b* mutants were growth deficient, and inviable at birth. Failures in gametogenesis and fertilization were seen, with the defects severer on ablation of *H3f3b*. Our results demonstrate important functions of H3.3 in growth, viability, and fertility, and reveal stages of gametogenesis and fertilization that are adversely affected by H3.3 deficiency in the mouse.

## Results

### *H3f3a* mutants are viable, with males slightly growth deficient

**Fig 1:**
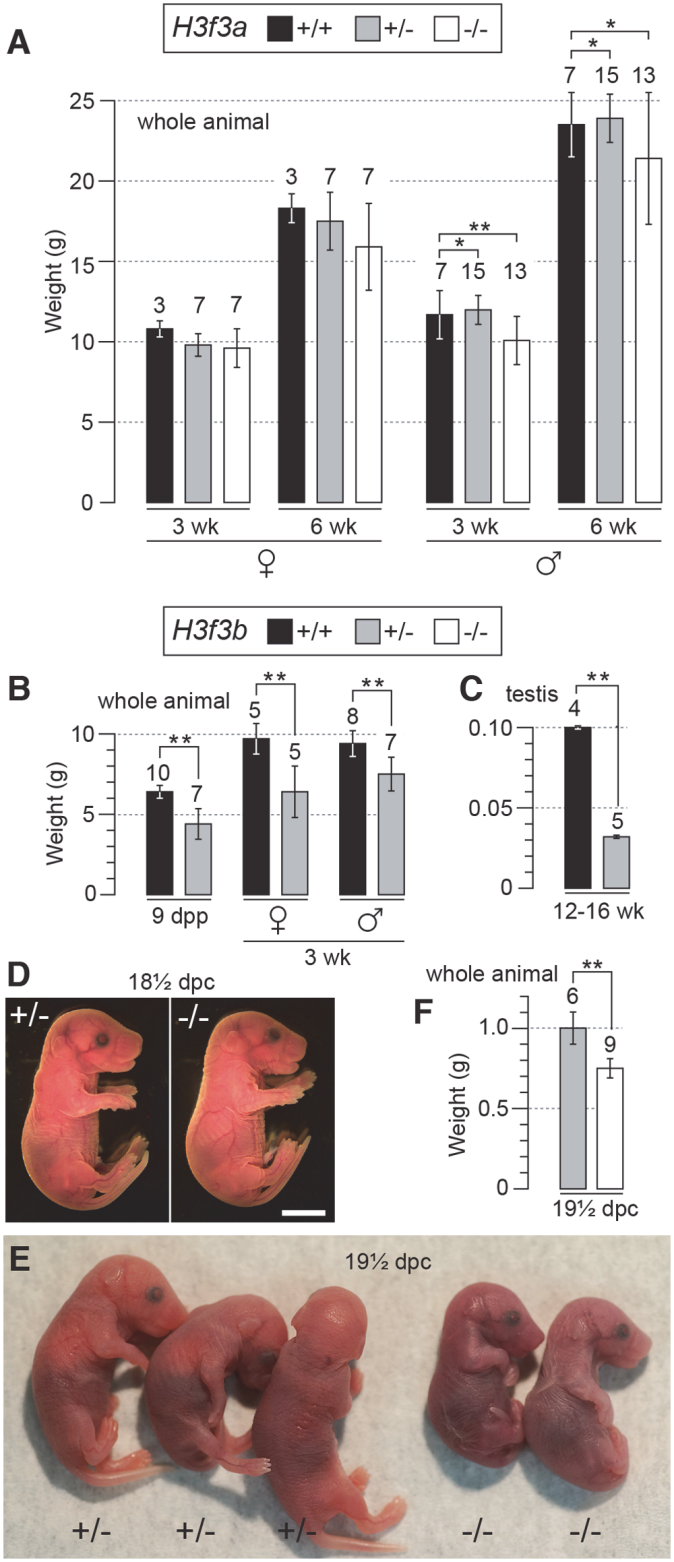
Pre- and postnatal developmental potential of mutants. (A) *H3f3a* mutants. Genotypes are shown in the box. Postnatal weight gain. Bars are mean ± s.d. with the numbers of animals weighed above the bar. (B) *H3f3b* mutants, postnatal weight gain, (C) *H3f3b* mutants, adult testis weight, (D) *H3f3b* mutants, two uterus-mates, two days before birth. Genotype of each is provided. Scale bar, 0.5 cm. (E) appearance, and (F) weight, of 19½ dpc *H3f3b* mutants delivered by Caesarean. Genotype of each is provided. *P<0.05, **P<0.01, *t*-test.

*H3f3a* ^+/-^ heterozygous young were produced by mating ES cell chimaeras to strain 129S1 Cre-deleter females [19] (see Materials and Methods). *H3f3a* ^+/-^ females and males were overtly normal and fertile. Timed matings were obtained from *H3f3a* ^+/-^ ♀ × *H3f3a* ^+/-^ ♂ intercrosses so that newborns could be monitored. In 14 litters in which 65 pups were born, only four dead pups were seen: two of these dead pups were *H3f3a* ^-/-^. All remaining young survived to adulthood, except two *H3f3a* ^-/-^ runts that were culled. From all timed pregnancies, and other intercrosses, 29, 84 and 46 animals surviving to adulthood were *H3f3a* ^-/-^ mutant, *H3f3a* ^+/-^ and *H3f3a* ^+/+^ (WT), respectively—not significantly different from a 1:2:1 ratio (*P*>0.05, chi-squared test). *H3f3a* ^-/-^ females were of normal size. By contrast, *H3f3a* ^-/-^ males were slightly smaller than *H3f3a* ^+/-^ and WT males at 3 wk and 6 wk (Fig. 1A). *H3f3a* ^-/-^ mutants of both sexes were overtly normal in behaviour and appearance.

### *H3f3b* heterozygotes are growth deficient, while *H3f3b* mutants die at birth

*H3f3b* ^+/-^ young were bred by mating ES cell chimaeras to strain 129S1 Cre-deleter females (see Materials and Methods). These young often appeared smaller than WT sibs at weaning, but developed into robust adults. *H3f3b* ^+/-^ females were fertile on mating with WT 129S1 males. From these matings, altricial and weaned *H3f3b* ^-/+^ young were significantly smaller than WT littermates, revealing haploinsufficiency for *H3f3b* (Fig. 1B).

*H3f3b* ^-/-^ mutants could not be bred in intercrosses because *H3f3b* ^-/+^ males were sterile, with small testes (Fig. 1C). To produce *H3f3b* ^-/-^ zygotes, we again made use of the 129S1 Cre-deleter line. The *cre* cds in this line is X-linked (X^*cre*^). Floxed alleles are always mutated on sperm entry into Cre-containing oocytes when XX^*cre*^ females are used [19]. Two sequential matings were performed. First, *H3f3b* ^-/+^, XX^*cre*^ females were bred in *H3f3b* ^c/c^, XX ♀ × WT, X^*cre*^Y ♂ matings—these females were converted to the *H3f3b* ^-/+^ genotype early in development because of the presence of X^*cre*^. Second, *H3f3b* ^-/+^, XX^*cre*^ ♀ × *H3f3b* ^c/c^, XY ♂ matings were performed to obtain timed pregnancies. The paternal *H3f3b* ^c^ allele was converted to *H3f3b* ^-^ in the zygote in these matings. Therefore, a 1:1 ratio of *H3f3b* ^-/-^ to *H3f3b* ^+/-^ zygotes was expected. Results of fetal recovery are shown (Table 1). Overall, 37 *H3f3b* ^-/-^ and 69 *H3f3b* ^+/-^ fetuses were obtained, different from a 1:1 ratio. Thirty-six deciduae were counted in these pregnancies, these being implantation sites in which development did not proceed beyond the early implantation stage. It is possible that most of these deciduae represented failures in *H3f3b* ^-/-^ development, accounting for the fetal deficit. This failure was not associated with sex chromosome constitution or to the presence of X^*cre*^ (Table 1).

**Table 1:** Survival of *H3f3b* mutant fetuses.

Age	*H3f3b* ^+/-^	*H3f3b* ^-/-^			Deciduae ^c^
	Total (inviable) ^a^	Total (inviable) ^a^	Y Chr ^b^	X^*cre*^ ^b^	
13½ dpc	39 (0)	23 (4)	11	9	28
16½ dpc	13 (2)	9 (3)	3	4	2
18½ dpc	17 (0)	5 (2)	4	2	6
Total	69 (2)	37 ^d^ (9)	18 ^d^	15 ^d^	36

^a^ Total number obtained, with the number inviable, i.e. dead or severely abnormal, in parentheses. Dead fetuses were scored when some amount of recognizable embryonic material was present.

^b^ Presence of the Y or X^*cre*^ chromosome (Chr) was determined by PCR assay.

^c^ Deciduae were scored as implantation sites that contained no discernible embryonic material.

^d^ Probability of a 1:1 ratio: Total, 37:69, *P*<0.01; Y chromosome, 18:19, *P*>0.05; X^*cre*^, 15:22, *P*>0.05; Fisher’s exact test.

Twenty four percent (9/37) of *H3f3b* ^-/-^ fetuses at 13½-18½ dpc were dead or severely abnormal (Table 1). Live 18½ dpc *H3f3b* ^-/-^ fetuses, two days before birth, had no gross abnormalities (Fig. 1D). However, no live *H3f3b* ^-/-^ pups were seen in newborn litters. Thirty two *H3f3b* ^+/-^ animals were born, 29 of which survived to weaning. A single *H3f3b* ^-/-^ dead pup was found on the day of birth. These results strongly suggest that all *H3f3b* ^-/-^ pups died at birth, then were consumed by the mothers. The immediate death of pups at birth is usually associated with respiratory failure [20]. Examining 18½ dpc *H3f3b* ^-/-^ fetuses histologically revealed no gross pathology in any tissues, including the lungs (S1 Fig.). To confirm that *H3f3b* ^-/-^ fetuses could not survive parturition, fetuses were delivered by Caesarean at 19½ dpc. From 5 pregnancies, of 11 *H3f3b* ^+/-^ fetuses delivered, all initiated normal respiration within a few minutes, remaining oxygenated or ‘pink’, and mobile. By contrast, all 6 *H3f3b* ^-/-^ fetuses delivered failed to initiate respiration after initially gulping. Within a few minutes they were immobile, and ‘blue’ (Fig. 1E). They were also smaller than *H3f3b* ^+/-^ littermates (Fig. 1F).

### *H3f3a* and *H3f3b* mutants contain similar amounts of residual H3.3 in somatic tissues

**Fig 2:**
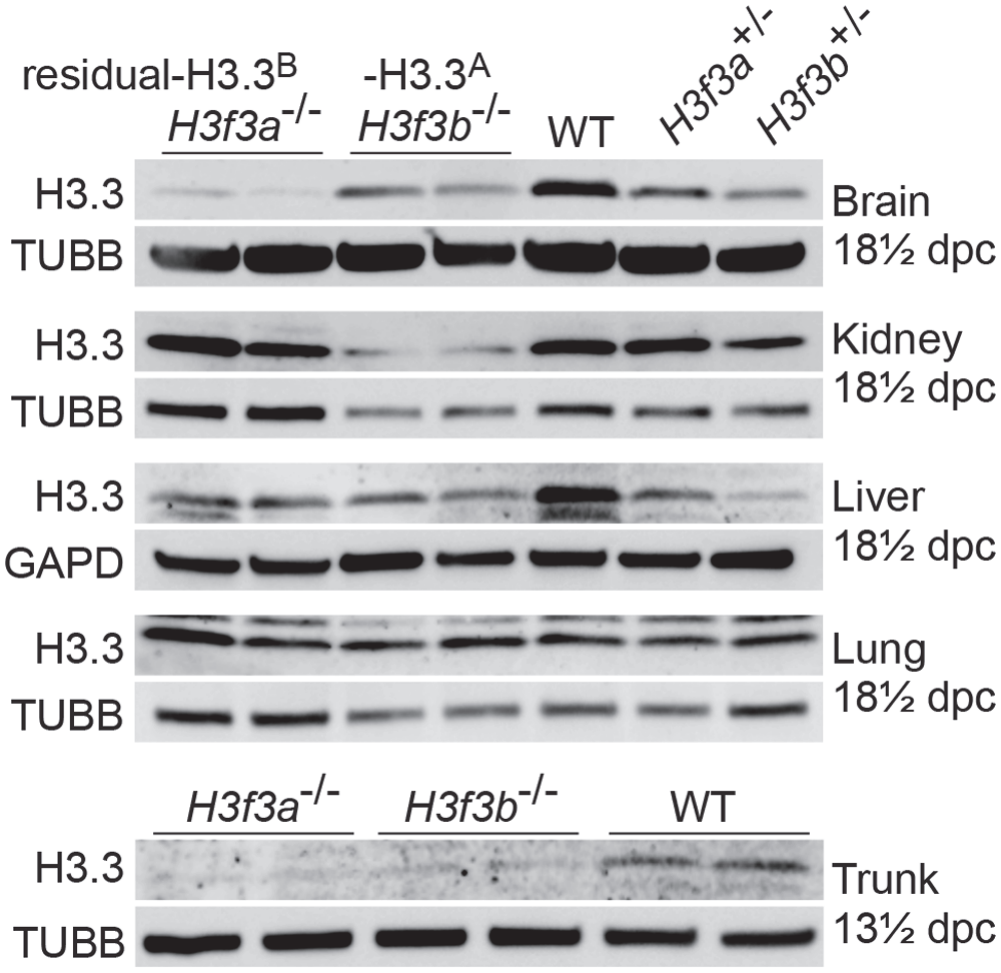
Relative amounts of residual H3.3^A^ and H3.3^B^ in fetal tissues. Immunoblots. Recombinant H3.3 was run alongside the samples to ensure the correct band was identified. In 13½ dpc fetuses, the trunk is the whole body minus the head. Tubulin-β (TUBB), and glyceraldehyde-3-phosphate dehydrogenase (GAPD) were used as loading controls.

*H3f3a* ^-/-^ mutants contain residual H3.3^B^, while *H3f3b* ^-/-^ mutants contain residual H3.3^A^. These amounts of residual H3.3 were determined in immunoblots in the brain, kidney, liver and lung of 18½ dpc fetuses, and in the trunk of 13½ dpc fetuses (Fig. 2). In both mutants, H3.3 was detected in all organs, consistent with H3.3^A^ and H3.3^B^ being essentially ubiquitous in the developing fetus. Given that *H3f3b* ^-/-^ mutants had the severer phenotype, we expected to see relatively less residual H3.3 in these animals. However, the only clear difference between the two mutants was in the brain, where *H3f3b* ^-/-^ animals had relatively higher residual H3.3^A^ (Fig. 2).

### *H3f3a* mutant males are subfertile

**Fig 3:**
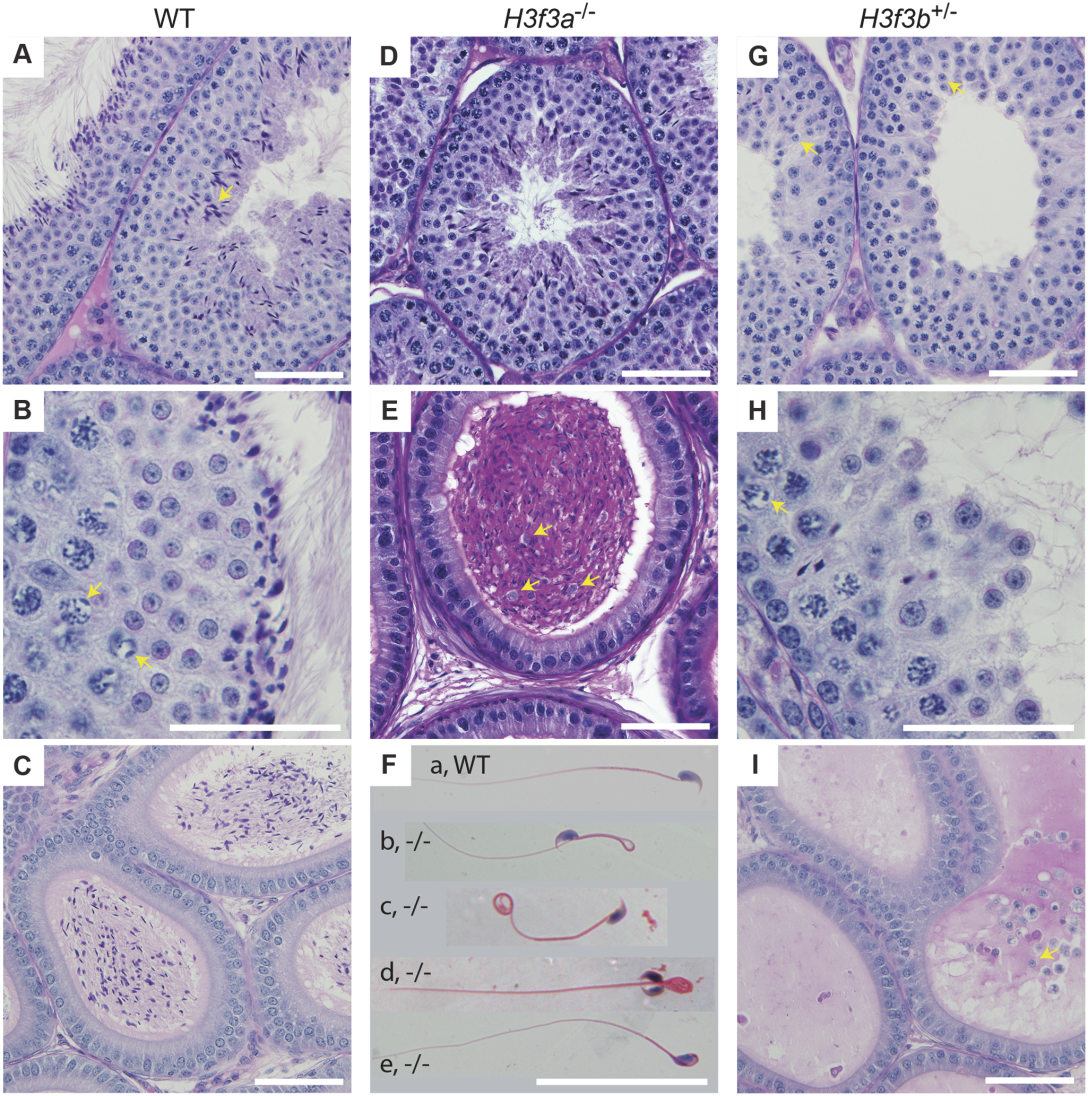
Testis and sperm morphology. WT adult, 6–10 months: (A) seminiferous tubule, condensing spermatid indicated (arrow), (B) seminiferous tubule, XY-body indicated (arrows), (C) cauda epididymis. *H3f3a* ^-/-^ adult, 6–10 months: (D) seminiferous tubule, (E) cauda epididymis, sloughed germ cells indicated (arrows), (F) spermatozoa, genotype as given; (a) normal, (b) bend back tail, (c) curly tail, (d) double head, (e) abnormal head. *H3f3b* ^-/+^ adult, 8 wk: (G) seminiferous tubule, arrested round spermatids (arrows), (H) seminiferous tubule, XY-body indicated (arrow), (I) cauda epididymis, sloughed spermatid indicated (arrow). Paraffin-embedded sections were PAS-stained. Scale bars, 25 μm.

**Fig 4:**
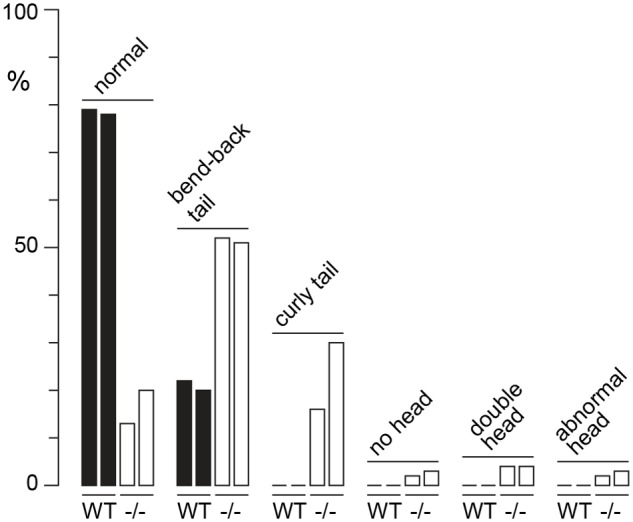
Sperm defects in *H3f3a* mutants. Defects were scored in sperm spreads made from two WT males at 6–10 months of age, and two age-matched subfertile *H3f3a* ^-/-^ males. More than 200 sperm were scored for each of the four males, with the prevalence of defects expressed as a percent of the total scored.

Three *H3f3a* ^-/-^ males were fertility-tested alongside three WT littermates within the first three months after reaching adulthood. Each male was caged for one week with two nulliparous 8–10 wk outbred Swiss females, this being repeated over several weeks. For *H3f3a* ^-/-^ males, 7 pregnancies from 34 female exposures resulted, less than the frequency for WT males—16 pregnancies from 28 exposures (*P*<0.001, Fisher’s exact test). Also, litters sired by *H3f3a* ^-/-^ males were small: WT, 10.63 ± 2.55 (16); *H3f3a* ^-/-^, 6.14 ± 4.85 (7) [mean ± s.d. (n)] (*P*<0.01, *t*-test). These three littermate pairs, and two additional pairs, were then fertility-tested at 6–10 months of age. Over this period, each male was caged with two Swiss females. All WT males consistently sired large litters, while three *H3f3a* ^-/-^ males sired no litters, and two *H3f3a* ^-/-^ males sired the occasional small litter. At the end of this period, the ability of *H3f3a* ^-/-^ males to copulate was tested. Vaginal plugs, at ½ days post coitum (dpc), were readily obtained when *H3f3a* ^-/-^ males were mated with nulliparous Swiss females. Oviducts were flushed at 1½ dpc. *H3f3a* ^-/-^ males fertilized substantially fewer ova than WT littermates, indicating that the subfertility was caused by a defect in spermatogenesis (Table 2). The reproductive tracts were overtly normal, except the vasa deferentia appeared to be devoid of sperm. Representative histological sections of the testes and caudae epididymides are shown (Fig. 3 A–E). A substantial production of sperm was seen in *H3f3a* ^-/-^ males, although some sloughed germ cells were present in the caudae, indicating developmental failure of some spermatids (Fig. 3D, E). Sperm isolated from the caudae showed reduced motility, failing to efficiently swim out in media. The large majority of spermatozoa possessed tail defects, with some possessing head defects (Figs. 3F, 4). These data are consistent with a requirement for H3.3^A^ in spermiogenesis.

**Table 2:** Fertilization frequency obtained in aged *H3f3a* mutant males.

Littermate pair	No. fertilized ova ^a^/unfertilized ova (no. plugs)	
	WT	*H3f3a* ^-/-^
1	39/39 (3)	0/63 (5)
2	24/24 (2)	0/55 (5)
3	n.d. ^b^	4/48 (4)
4	37/83 (8)	1/73 (7)
5	49/59 (5)	18/43 (4)
Total	149/205 (73%)	23/282 (8%)**

^a^ scored as 2-cell ova

^b^ not done, mouse died prior to testing

Probability of *H3f3a* ^-/-^ being equal to WT:

**P<0.01, Fisher’s exact test.

### *H3f3b* heterozygous males are sterile, with developmental arrest of round spermatids

*H3f3b* heterozygous males derived from chimaeras mated to Cre-deleter females, or from the *H3f3b* ^+/-^ ♀ × WT ♂ backcross, were sterile. Their testes were less than one third of normal weight, indicative of failed spermatogenesis (Fig. 1C). Histological analysis revealed a reduction of round spermatids and very few elongating spermatids in the lumen of seminiferous tubules (Fig. 3G, H). The caudae epididymides were devoid of sperm, and often contained sloughed germ cells (Fig. 3I).

**Fig 5:**
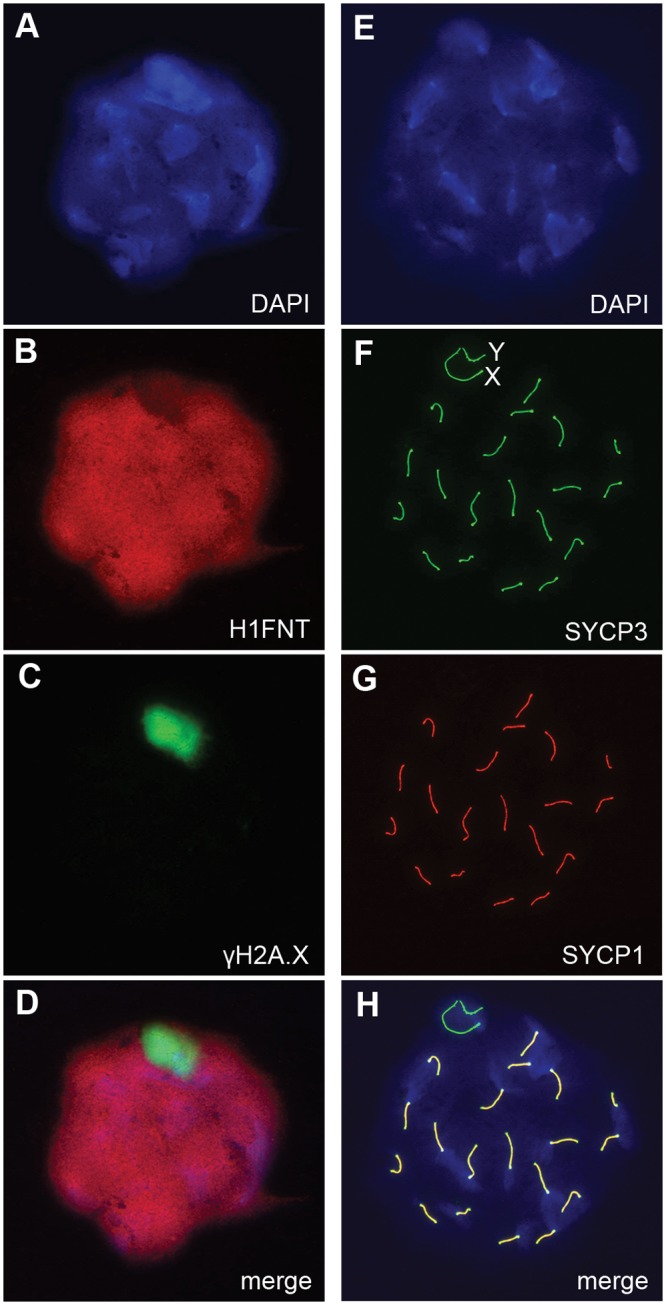
Autosomal synapsis in *H3f3b* heterozygous spermatocytes. *H3f3b* ^-/+^ male, 3 wk old. Spermatocyte 1: (A) DNA, DAPI stained, (B) mid-late pachytene spermatocytes are H1FNT-positive, (C) γH2A.X is localized to the XY-body. No γH2A.X staining evident in autosomes, indicative of normal synapsis, (D) merge of the three upper panels. Spermatocyte 2: (E) DNA, DAPI stained, (F) SYCP3 marks the chromosomal axial elements. By pachytene, these have coalesced into rod-like structures, (G) SYCP1 marks synapsed chromatin. The X and Y chromosomes are unsynapsed, therefore are negative for this marker, (H) merge of the three upper panels shows that SYCP3 and SYCP1 are coincident, indicating complete synapsis.

H3.3 has been reported to preferentially localize to the XY-body, where it may play a role in MSCI [15]. Therefore, we thought there may a level of MSCI failure in *H3f3b* ^-/+^ males. MSCI acts as a checkpoint. Its failure is associated with autosomal asynapsis, leads to pachytene apoptosis with no progression of spermatocytes beyond meiosis-I [21, 22]. The presence of pachytene spermatocytes with XY-bodies, and round spermatids in *H3f3b* ^-/+^ sterile males (Fig. 3H), indicates that there is no serious failure of MSCI. Yet, we decided to investigate autosomal synapsis in *H3f3b* ^-/+^ males more directly. First, we examined the localization of γH2A.X (H2A histone family, member X) in mid- to late-pachytene spermatocytes of *H3f3b* ^-/+^ males, when autosomes should be fully synapsed. These stages were marked by positive staining for H1FNT (H1 histone family, member N, testis-specific) [23]. All *H3f3b* ^-/+^ H1FNT-positive cells examined (>50) displayed discrete localization of γH2A.X to the presumptive XY-body, with no labelling evident outside this region, indicating that autosomal synapsis and MSCI proceeded normally (Fig. 5A–D). Complete autosomal synapsis in *H3f3b* ^-/+^ males was confirmed in double-staining for SYCP3 and SYCP1 (synaptonemal complex proteins 3 and 1). SYCP3 marks chromosomal axial elements that have assembled in meiosis-I, and is present before and during synapsis, while SYCP1 marks synapsed chromatin. All spreads with 20 discrete SYCP3-positive axial elements examined (>30) were equivalently positive for SYCP1, demonstrating complete synapsis. The unsynapsed sex chromosomes remained SYCP1-negative (Fig. 5E–H). Finally, no increase in apoptosis was seen in spermatocytes or round spermatids by TUNEL staining (S2 Fig.). Overall, these data strongly suggest that meiosis-I is essentially normal in *H3f3b* ^-/+^ spermatocytes. Thus, the failure in spermatogenesis is localized to round spermatids, which appear to arrest through mechanisms that do not involve apoptosis.

**Fig 6:**
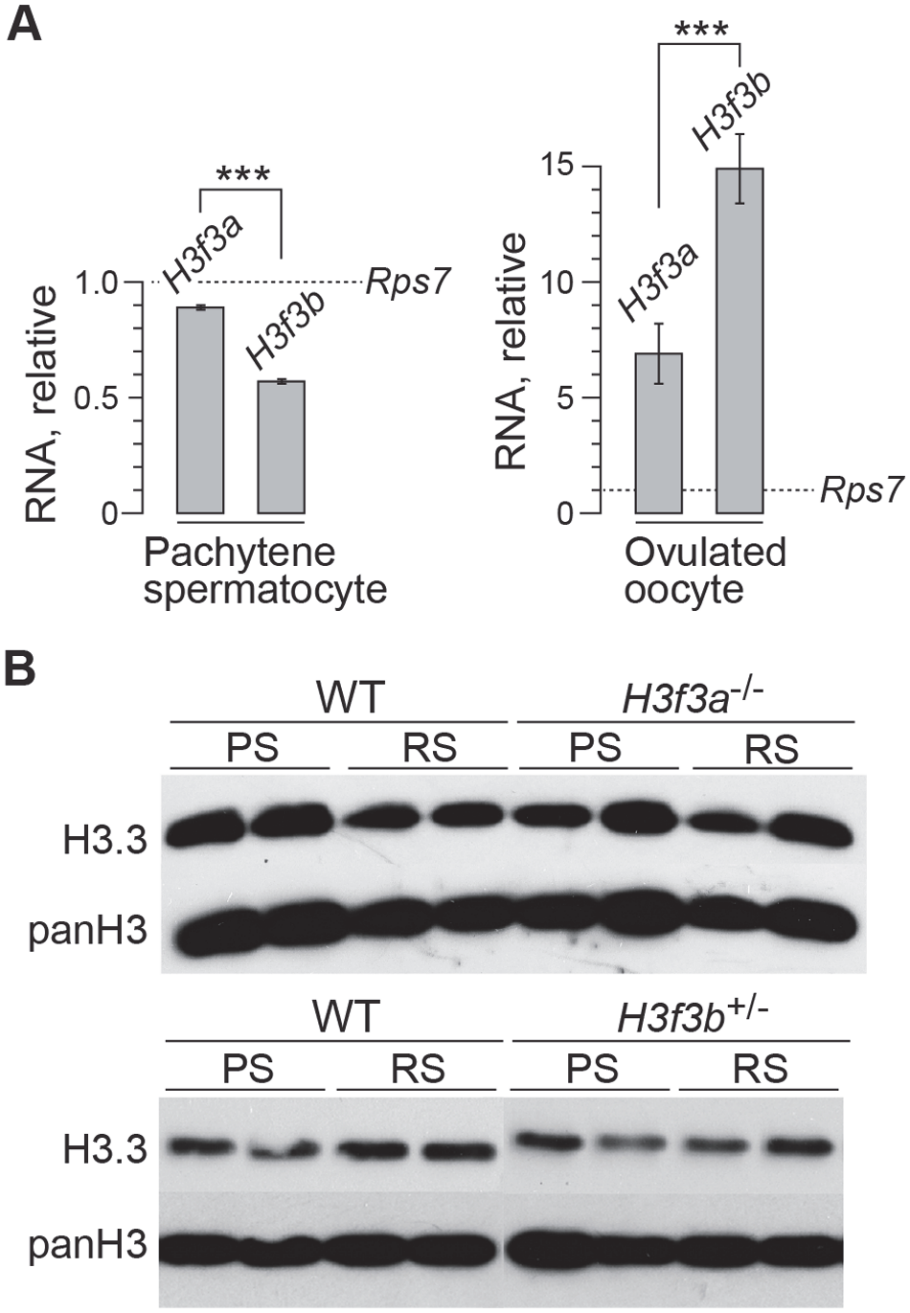
*H3f3a* and *H3f3b* RNA and H3.3 protein content in gametogenesis. (A) Quantitative PCR assays. Values for *H3f3a* and *H3f3b* RNA are relative to those obtained for the housekeeper *Rps7*, set at 1.0. ***P<0.001, *t*-test. (B) Immunoblots. H3.3 content in pachytene spermatocytes (PS) and round spermatids (RS). panH3, total H3.

These data suggest a crucial role for H3.3^B^ in early spermiogenesis, one that is relatively more important than for H3.3^A^. In *H3f3a* mutants, spermiogenesis was also abnormal, but did lead to the formation of sperm. RNA in situ hybridization analysis has shown that the expression of *H3f3b* is restricted to primary spermatocytes, while the expression of *H3f3a* is ubiquitous across spermatogenic cell types [24]. It therefore appears that the requirement for H3.3^B^ in spermiogenesis is fulfilled by translation in meiosis-I. The difference in severity of phenotype was not reflected in the relative amount of RNA, as we measured more *H3f3a* than *H3f3b* RNA in pachytene spermatocytes (Fig. 6A). Also, the amounts of H3.3 detected in pachytene spermatocytes and round spermatids of *H3f3a* ^-/-^ and *H3f3b* ^+/-^ males were not discernibly different to WT (Fig. 6B).

### *H3f3a* mutant females are fertile

*H3f3a* ^-/-^ females were of normal fertility. Of eight 6 wk females caged with WT 129S1 males, seven gave birth within 10 wk. Litter size was 4.86 ± 1.86 (7) [mean ± s.d. (n)], not significantly different to that obtained with *H3f3a* ^+/-^ females in intercrosses; 5.54 ± 2.54 (14) (*P*>0.05, *t*-test). *H3f3a* ^-/-^ females raised their young normally. Three *H3f3a* ^-/-^ second-generation female young, bred in *H3f3a* ^-/-^ ♀ × *H3f3a* ^-/-^ ♂ matings, were also fertile: when mated to a WT outbred Swiss male, each had three sequential litters no more that one month apart, and with no fewer than five pups in any litter. All pups were raised normally. These results show that H3.3^A^ is dispensable in the female germ line, and for fertilization and development.

### *H3f3b* mutagenesis at the beginning of folliculogenesis results in severe female subfertility

**Fig 7:**
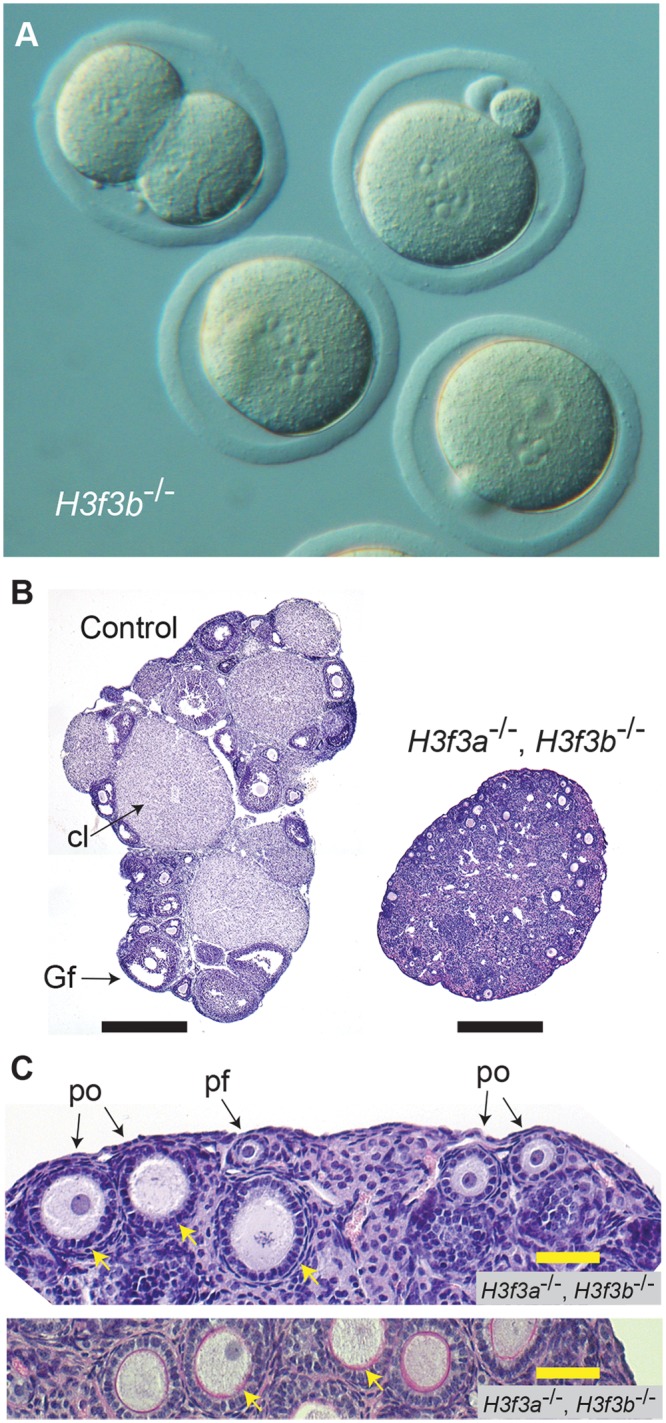
Developmental potential of *H3f3b* mutant zygotes, and *H3f3a*, *H3f3b* double-mutant follicles. (A) Presumptive *H3f3b* ^-/-^ ova were flushed from the oviducts of experimental females at 1½ dpc. Some 2-cell ova were obtained, with one shown at top-left. (B) left panel, ovary of an experimental *H3f3a* ^c/c^, *H3f3b* ^c/c^, 0/Tg(Zp3-cre) female at 10 wk, in which oocytes are of presumptive genotype *H3f3a* ^-/-^, *H3f3b* ^-/-^; right panel, control 10 wk ovary showing prominent Graafian follices (Gf), and copora lutea (cl). Bars, 0.5 mm, (C) top panel, 10 wk experimental ovary showing resting primordial follicles (pf), and early-stage growing primary oocytes (po). Degenerating primary oocytes are indicated (arrows); bottom panel, experimental 10 wk ovary stained with PAS to show the zona pellucida (pink, arrows). Scale bars, 50 μm.

To determine the requirement for H3.3^B^ in folliculogenesis, we used the *Zp3* (zona pellucida protein 3) promoter-*cre* cds transgenic mouse line, Tg(Zp3-cre), which excises floxed sequences at primordial follicle activation [25, 26]. We bred a Tg(Zp3-cre) congenic line in strain 129S1 to perform all the experiments described. *H3f3b* ^c/c^, 0/0 ♀ × *H3f3b* ^c/c^, 0/Tg(Zp3-cre) ♂ matings produced control *H3f3b* ^c/c^, 0/0, and experimental *H3f3b* ^c/c^, 0/Tg(Zp3-cre) females. These females were mated with WT males to obtain timed pregnancies. Less *H3f3b* ^c/c^, 0/Tg(Zp3-cre) or experimental females became pregnant, and in these, fetal recovery was very low—only 6% in successful matings, relative to control females (Table 3). A few females were allowed to give birth, and pups were viable to adulthood. No deciduae were observed in any of the pregnant or non-pregnant plugged experimental females, indicating that there was a failure in ovulation, fertilization, or cleavage. Ova were scored at ½ dpc. The number obtained in control and experimental female matings was equivalent. However, fewer ova in experimental females were fertilized (Table 3). Zygotes, with visible pronuclei, were cultured overnight to 1½ dpc: only 10% of experimental pronuclear zygotes could cleave. Cleavage failure was also observed in vivo, when oviducts of experimental females were flushed at 1½ dpc (Table 3). Experimental zygotes that failed to cleave are shown (Fig. 7A).

**Table 3:** Developmental potential of *H3f3b* mutant oocytes.

Genotype ^a^	Frequency of					
	pregnancy ^b^	fetuses ^c^	ovulation ^d^	fertilization ^e^	Cleavage (in vitro) ^f^	Cleavage (in vivo) ^g^
c/c, 0/0	8/8 (100%)	7.88 ± 1.25 (8)	9.09 ± 1.04 (11)	98/100 (11) (98%)	48/50 (5) (96%)	38/39 (4) (97%)
-/-, 0/cre	4/11 (36%)**	1.25 ± 0.50 (4)**	9.62 ± 1.12 (13)^NS^	90/125 (13) (72%)**	6/62 (7) (10%)**	13/49 (7) (27%)**

^a^ *H3f3b*, Tg(Zp3.cre).

^b^ no. females pregnant/total plugged females (%).

^c^ no. fetuses per pregnancy [mean ± s.d. (n)].

^d^ mean no. of ova per plugged female [mean ± s.d. (n)].

^e^ no. ova fertilized/total recovered (%).

^f^ no. zygotes with pronuclei cleaved overnight to 2-cell/total recovered (%).

^g^ no. 2-cell ova recovered at 1½ dpc/total recovered—non-cleaved ova with pronuclei (%).

Probability of the two genotypes being the same:

NS, not significantly different,

**P<0.01, *t*-test or Fisher’s exact test.

**Fig 8:**
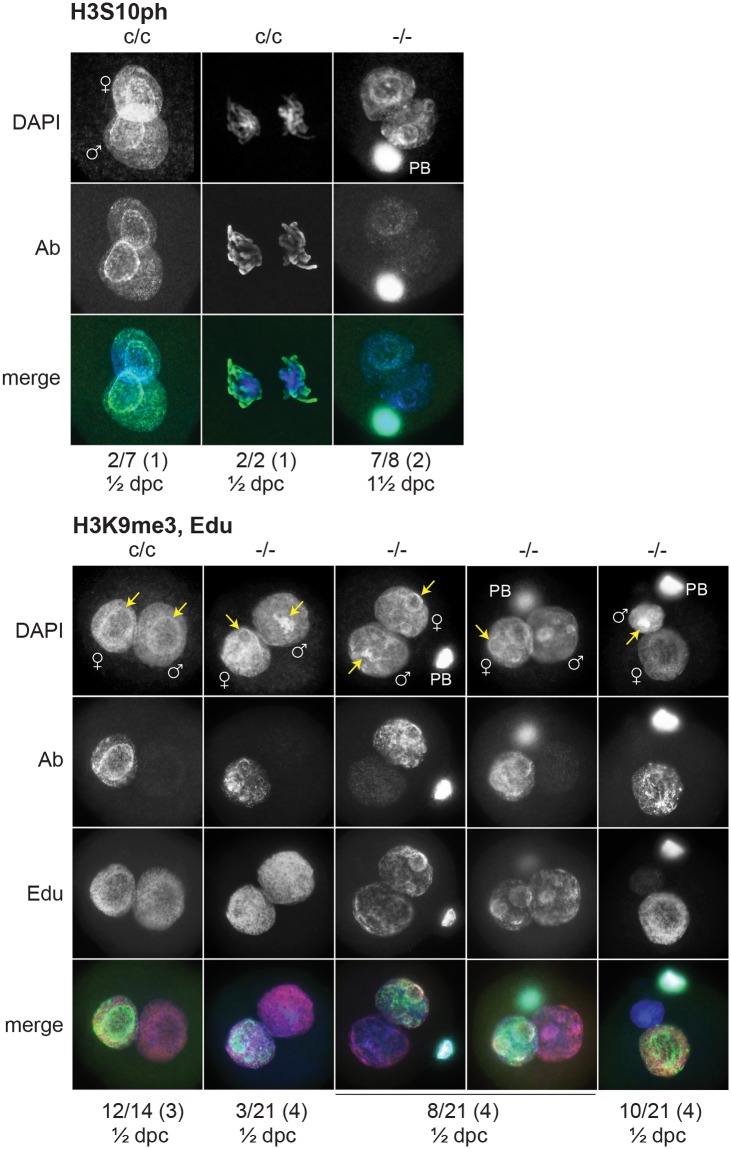
Chromatin analysis in *H3f3b* mutant zygotes. c/c, *H3f3b* ^c/c^ control zygotes from Tg(Zp3.cre)-negative females mated to WT males; -/-, *H3f3b* ^-/-^ experimental or mutant zygotes from Tg(Zp3.cre)-positive females mated to WT males. Under the plates: number of zygotes with staining as shown above/total observed (number of plugged females); ½ dpc, ampullae dissected on day of vaginal plug; 1½ dpc, ampullae dissected on the day after the vaginal plug. Nucleolar precursor bodies are indicated (arrows).

To gain insight into the cause of cleavage failure in *H3f3b* ^-/-^ zygotes, we studied aspects of chromatin structure using immunofluorescence. A hallmark of late prophase and mitosis is the serine phosphorylation of histone H3 (H3Sph). The deposition dynamics of this mark are highly conserved, suggestive of important roles in mitosis. Indeed, it has been recently shown that H3S10ph is required for proper chromosome condensation in budding yeast (*Saccharomyces cerevisiae*) [27]. *H3f3b* ^-/-^ zygotes did not accumulate this mark, even after overnight culture, indicating that they failed to progress to late prophase (Fig. 8). Staining for H3K9me3, being specific for the maternal pronucleus [28, 29], revealed that the paternal pronucleus was often similar in size or smaller than the maternal, instead of being consistently larger, as in controls (Fig. 8). We also typically observed small and dispersed nucleolar precursor bodies (NPBs) in the maternal pronuclei of *H3f3b* ^-/-^ zygotes—evident under DAPI staining. Control *H3f3b* ^c/c^ zygotes typically contained one large NPB in each pronucleus (Fig. 8). Similarly, the central regions of intense DAPI staining in the paternal pronuclei of *H3f3b* ^-/-^ zygotes could be related to a reduction in the development of NPBs: normally, intense DAPI staining is concentrated at the periphery of NPBs, corresponding to constitutive heterochromatin [29, 30]. DNA replication was examined in *H3f3b* ^-/-^ zygotes using Edu incorporation, concomitant with H3K9me3 immunofluorescence so that the maternal and paternal pronuclei could be definitively discerned. Three classes of *H3f3b* ^-/-^ zygote were seen in respect to relative pronuclear size and DNA synthesis: (1) paternal larger than the maternal, robust replication in both—3 of 21 zygotes, (2) paternal similar to the maternal, reduced replication in at least the paternal—8 of 21 zygotes, and (3) paternal smaller than the maternal, replication in only the maternal—10 of 21 zygotes (Fig. 8). Thus, all mutant zygotes appeared to have smaller NPBs, while most showed reductions in DNA replication.

### *H3f3a*, *H3f3b* double-mutagenesis in folliculogenesis results in primary oocyte failure

The developmental potential of *H3f3a* and *H3f3b* mutant oocytes as described above, indicates that *H3f3b* is either the predominant source, or the only source, of H3.3 during folliculogenesis. To distinguish between these possibilities, we first examined the relative amounts of *H3f3a* and *H3f3b* RNA in ovulated unfertilized oocytes by quantitative real-time PCR. Both RNAs are present, with the amount of *H3f3b* RNA being double that of *H3f3a* (Fig. 6A). Next, we assessed the developmental potential of double-conditional experimental *H3f3a* ^c/c^, *H3f3b* ^c/c^, 0/Tg(Zp3-cre) oocytes, in which H3.3^A^ and H3.3^B^ should be eliminated soon after primary oocyte activation. Presumptive double-mutant primary oocytes were inviable. Experimental females at 4 wk had very small ovaries, with no apparent oocyte growth. A similar phenotype was seen in 10 wk experimental females (Fig. 7B). Primordial follicles, of ~11 μm in diameter, and very early-stage primary oocytes, were of normal appearance. By contrast, nearly all primary oocytes developing a second layer of follicle cells were degenerating, reaching a maximum of ~50 μm in diameter (Fig. 7C). Very few larger primordial oocytes were seen, except for a rare early-stage antral follicle. This result indicates that *H3f3a* and *H3f3b* are both sources of H3.3 during folliculogenesis, and clearly shows that H3.3 is absolutely required. Despite the inviability of double-mutant primary oocytes, they had undergone a significant development of the zona pellucida, indicating that the genes encoding the zona glycoproteins were being actively transcribed up to the stage of oocyte death (Fig. 7C).

## Discussion

We have assessed the contribution to viability and fertility of the two unlinked genes encoding the histone variant H3.3 in mice. The findings provide implications for the relative importance of each gene. *H3f3a* and *H3f3b* mutants could develop to late gestation, demonstrating no absolute requirement for either gene up to this stage. *H3f3a* is strongly ubiquitously expressed during development [31, 32], and the present study indicates this is also true for *H3f3b*. Both genes have CpG-island promoters, consistent with housekeeper expression. Given the wide expression profile of each gene, a level of functional redundancy is likely. The co-ablation of *H3f3a* and *H3f3b* RNA in zygotes by morpholinos resulted in morula-stage arrest, revealing functional importance of H3.3 at this early stage [33]. It remains of interest to determine the developmental potential of double-mutant animals in our experimental system. Double-mutants may survive the preimplantation period because of the presence of maternal *H3f3a* and *H3f3b* RNAs. Also, mouse ES cells in which H3.3 is depleted are viable, and are able to differentiate into all three germ layers in teratomas [9].

The presence of two H3.3-encoding genes appears to be important, given its conservation in at least mammals and birds [18]. This gene doubling may be necessary to satisfy the high demand for this protein in DNA packaging and other downstream functions, akin to the multiplicity of canonical H3.1- and H3.2-encoding genes. Sequences outside the cds of *H3f3a* and *H3f3b* are nevertheless highly divergent. From the present results, *H3f3b* is generally functionally more important than *H3f3a*, given *H3f3b* mutants were more severely affected than *H3f3a* mutants. This was seen in fetal and postnatal growth, perinatal survivability, spermiogenesis, and zygote development. However, this conclusion assumes that there is no compensatory regulation between the two genes. This cannot be examined at the protein level in WT animals, as H3.3^A^ and H3.3^B^ cannot be distinguished. Surprisingly, *H3f3a* and *H3f3b* mutants contained similar amounts of residual H3.3 in a range of tissues examined, except brain, where the residual H3.3^A^ was higher. These findings indicate that differences in expression of the two genes at the sub-organ tissue-specific level are the most important determinants of the relative functional importance of H3.3^A^ and H3.3^B^. Differences in tissue-specificity of expression is suggested in the recent findings that paediatric brain tumors, and giant cell tumors of bone, contain driver amino acid substitution mutations in *H3F3A*, while chondroblastomas contain driver mutations in *H3F3B* [16, 17]. A detailed investigation of the tissue-specific expression of each of these genes in development would be of interest.

Approximately one half of *H3f3b* mutants appeared to die at the implantation or early postimplantation stage. The other half developed to fetal stages, with the majority reaching birth. One possible explanation for this developmental bipotentiality is chromosomal instability. A higher level of aneuploidy was reported in *H3f3b* mutant primary embryo fibroblasts [34], therefore there could exist a potential for aneuploidy at the preimplantation stage. Most aneuploid blastocysts die at implantation, or shortly thereafter [35]. The failure of *H3f3b* mutants to initiate respiration at birth was not accompanied by any lung histopathology, but there could be lung surfactant or neural deficits that lead to immediate respiratory failure [20].

The defects induced by H3.3^B^ deficiency in the germ line were striking. The constitutive loss of one *H3f3b* allele led to the arrest of round spermatids, and sterility. By contrast, the loss of both *H3f3a* alleles resulted in dysmorphic spermatozoa, and subfertility. This difference in severity occurred despite our lack of evidence that *H3f3b* is expressed at a higher level than *H3f3a*, or is the major source of H3.3, in pachytene spermatocytes and round spermatids. This raises the possibility that there could be qualitative differences in the distribution of H3.3^A^ and H3.3^B^ in spermatogenic cells. Differential expression of the two genes during the prolonged meiotic cycle could lead to regional and functional differences in the incorporation of each protein into chromatin, depending on what sites are vacant for incorporation, and when.

The phenotypes described here in *H3f3a* and *H3f3b* mutants differ substantially from phenotypes described in other studies. A hypomorphic gene-trap mutation in *H3f3a* led to very high mortality by weaning [36]. In explaining our milder phenotype, genetic background is an unlikely explanation, as both *H3f3a* mutations were propagated in a ‘Steel’ substrain of strain 129 [37]. Environmental influences are a possible explanation: the survivability of *H3f3a* mutants to weaning was increased when litter size was reduced [36]. Finally, the *H3f3a* retroviral gene-trap mutation was reported to produce up to ~20% normal levels of full length *H3f3a* RNA [36]. Therefore, retroviral sequences could have resulted in a deregulation of residual H3.3^A^ that was detrimental to viability.

In contrast to the situation for *H3f3a*, our *H3f3b* mutants were considerably more affected than those previously described [34, 38]. In this earlier study, a normal frequency of *H3f3b* mutants was obtained at midgestation, although most were substantially growth retarded. Despite this prominent defect, many mutants survived parturition and developed to adulthood. No growth deficit was seen in postnatal *H3f3b* heterozygous or mutant animals [34]. By marked contrast, our *H3f3b* mutants could not survive beyond birth, and a significant number died even before this stage. Further, we saw marked reductions in growth in perinatal and postnatal heterozygous and mutant animals.

The greater phenotypic severity associated with our *H3f3b* mutation was also seen in the male germ line. We saw a near-uniform arrest of round spermatids in heterozygous males. By contrast, previously reported heterozygous males were fertile [34]. Indeed, the phenotypic severity in our heterozygous animals exceeded even that seen in recently described *H3f3b* mutants, where appreciable progression through spermiogenesis was observed. These mutants were sterile due to the production of sperm with motility and head defects [38]. Other differences were apparent. We saw no increase in apoptosis by TUNEL staining in seminiferous tubules of heterozygotes, while an increase was seen in mutants [38]. Reduced protamine loading was reported in mutants, and this could be a major contributor to the defective head formation in mature sperm [38]. This phenotype was similar to that seen in our *H3f3a* mutant males, therefore there could be a similar mechanistic basis. However, in our *H3f3b* heterozygous males, other explanations are required, as the stage of arrest precedes the phase of histone displacement. In round spermatids, there is a large increase in transcriptional activity [39,40], and defects in this transcriptional activation could lead to their arrest. The simplest explanation for the differences in effect of the two *H3f3b* mutations is genetic background. Ours was kept in strain 129S1, while the other was kept in strain C57BL/6 [38]. A caveat in interpreting the present, and previous, results is that the *H3f3b* mutations were constitutive. H3.3^B^ deficiency in Sertoli cells, which support spermatogenesis, could exacerbate the germ cell defects observed.

H3.3^B^ deficiency in folliculogenesis led to some fertilization failure, and zygotic arrest, demonstrating that *H3f3b* is a maternal effect gene. Loss of maternal Hira, a H3.3 chaperone, leads to zygote failure in flies (*Drosophila melanogaster*). After sperm entry, H3.3 fails to incorporate into paternal chromatin, leading to failed decondensation of sperm chromatin [41]. A similar effect has recently been described in mouse, where depletion of HIRA in folliculogenesis using the Tg(Zp3.cre) transgene resulted in zygote arrest. The loss of HIRA led to a failure of paternal pronucleus formation. Also, the loss of DNA replication and ribosomal RNA transcription in paternal and maternal chromatin was observed, and either one of these deficiencies can lead to zygote arrest [42]. The phenotype in our H3.3^B^-deficient mutant oocytes was similar to, but less severe than, the HIRA-deficient phenotype, and could be explained by the presence of residual H3.3^A^. Our phenotype was variable, ranging from reduced, to overtly normal, paternal pronuclear expansion. Only a small number of mutant zygotes showed robust DNA replication in both pronuclei. If these represent the few zygotes able to cleave, then incomplete DNA replication could be the predominant cause of failed entry into first mitosis. Another possible explanation is a deficiency of H3Sph, given the importance of H3.3 in DNA packaging in the zygote, and the probable requirement for H3Sph in chromosome condensation at the end of prophase [27]. This could be investigated by determining the developmental potential of H3.3^B^-deficient zygotes supplemented with mutant H3.3 lacking relevant serines. H3.3 incorporation has also been recently shown to be required for pronuclear pore formation [43] and it is possible that a defect in this assembly could explain the mitosis failure.

An important requirement for H3.3 during the early phase of oocyte growth was seen in the developmental failure of *H3f3a*, *H3f3b* double-mutant primary oocytes. Given that HIRA-deficient oogonia can develop and be ovulated, this result clearly demonstrates a requirement for other H3.3 chaperones, possibly DAXX and ATRX, in regulating folliculogenesis. Conditional mutagenesis of these chaperones during folliculogenesis could be informative. The death of H3.3-deficient primary oocytes could be related to the lack of incorporation by a chaperone aside from HIRA, or to the combined lack of incorporation by two or more chaperones.

This study provides insight into the relative importance of the two genes encoding H3.3 for development and fertility in the mouse, and provides a basis for further investigations into the various biological roles of this basic building block of chromatin.

## Materials and Methods

### Mouse lines

The mouse lines carrying Cre/*loxP* conditional mutant alleles of *H3f3a* and *H3f3b*, termed *H3f3a* ^c^ and *H3f3b* ^c^, have been described [32]. The Cre-deleter line used to derive mice carrying constitutive mutant alleles, termed *H3f3a* ^-^ and *H3f3b* ^-^, was 129S1/Sv-*Hprt* ^*tm1(cre)Mnn*^/J (Jackson Laboratory, stock no. 004302).

### Histology

Testes and ovaries from adult mice were fixed in Bouin’s fixative. Sections were embedded in paraffin and stained by Periodic-Acid Schiff (PAS). Fetuses at 18½ dpc were decapitated, transferred to 20 mL of Bouin’s fixative, rocked slowly for 3 days, then washed in four changes of 70% (v/v) ethanol over 2 days. Sections were stained with haematoxylin and eosin. Spreads of mature sperm were made by squeezing the cauda epididymis with the back of curved forceps for release into 0.3 mL of medium M2 [44]. Most of the droplet was transferred to a tube with a wide-bore yellow tip, then an equal volume of 10% phosphate buffered formalin added, and gently mixed. After fixation (15 min, RT), a small drop of the sperm suspension was smeared onto a slide, air-dried, and stained with haematoxylin and eosin.

### Preparation of spermatocyte and sperm spreads, and immunofluorescence

Spermatocyte spreads from 18–22 days post partum mice and immunofluorescence (IF) were performed as described [45], with the following modifications: both testes of an 18–22 days post partum (dpp) male were transferred to 2 mL of Dulbecco’s phosphate buffered saline (without calcium and magnesium, with 5.6 mM glucose and 0.4 mg/mL bovine serum albumin), in a petri dish, the tunicae removed, and cut into small pieces with scissors. The pieces were transferred to 3 mL of 0.25% trypsin-EDTA (Gibco, 25200) in a polystyrene tube (Falcon, 352054), incubated (37°C, 20 min), and triturated vigorously with a transfer pipette (30 sec). The crude suspension was passed through a cell strainer (Falcon, 352350) into 1 mL of fetal bovine serum in a 15 mL centrifuge tube, and mixed. The spermatocytes were pelleted (250 × *g*, 4 min), the pellet resuspended in 3 mL of medium M2, and the tube placed on ice. Protease inhibitors (Sigma Aldrich, P8340) were added to all of the solutions.

To prepare the spreads, 50 μL of the sperm suspension was combined with 0.1 mL of medium M2, and pelleted (500 × *g*, 2 min). The supernatant was removed, and the pellet resuspended in 40 μL of alkaline sucrose solution [45]. Two drops of this suspension were then dispensed from a yellow tip into ~0.1 mL of the 1% (w/v) paraformaldehyde fixative solution, this being previously dispensed within a rectangle of ~1.5 cm^2^ drawn on a SuperFrost slide with a PAP pen. Spermatocytes were allowed to settle onto the slides in a humidified chamber (2 h, RT). Protease inhibitors were added to all solutions, except the fixative solution. After removing from the chamber, slides were rinsed in water, then in 0.004% (v/v) Photo-Flo 200 (Kodak, 14634510) in water, and air-dried. Slides were stored at -20°C until hybridization with Abs. Imaging was performed with a Imager.M1 microscope and AxioCam MRm camera (Carl Zeiss).

Primary Abs used, dilution factor: anti-γH2A.X, rabbit mcAb, 400× (Cell Signaling, 9718); anti-H1FNT, guinea pig pcAb, 250× (kindly provided by Mary Ann Handel); anti-SYCP3, mouse mcAb, 100× (Abcam, 97672); anti-SYCP1, rabbit pcAb, 200× (Abcam, 15090). Secondary Abs used, dilution factor: anti-rabbit IgG, goat, 1000×, AlexaFluor 488; anti-guinea pig IgG, goat, 1000×, AlexaFluor 594; anti-mouse IgG, goat, 1000×, AlexaFluor 488; anti-rabbit IgG, goat, 1000×, AlexaFluor 594 (Life Technologies, A-11008, A-11076, A-11001 and A-11012, respectively).

### Spermatocyte and round spermatid purification

Pachytene spermatocytes (larger 4c cells) and round spermatids (smaller 1c cells) were purified by flow cytometry as described [46]: testis suspensions were obtained as described above, except that DNAseI (Sigma Aldrich, D4527) was added to the trypsin digest at 5 U/mL, and the cell pellet resuspended in 1.5 mL of medium M2 containing 1 U/mL of DNAseI. The final suspension was incubated with Hoechst 33342 (Life Technologies, H3570) for gating on larger pachytene spermatocytes (4c) and smaller round spermatids (1c), and propidium iodide (Life Technologies, P3566) for gating on live cells (S3 Fig.). Cells were sorted into 0.3 mL of medium M2 in 1.5 mL centrifuge tubes using an Influx cell sorter (Becton Dickinson), then pelleted (2,500 rpm, 3 min), the supernatant removed, and stored at -80°C.

### Quantitative real-time PCR

RNA was isolated using TRI Reagent (Life Technologies, AM9738) according to the instructions. Pachytene spermatocytes (200,000) were purified from WT prepubertal 129S1 males by flow cytometry as described above. Oocytes (200) were obtained from WT superovulated prepubertal 129S1 females. The zona pellucida was removed using acid tyrode’s solution [47] before lysis in TRI Reagent. Two random-primed reverse transcription (RT) reactions were performed with SuperScript III Reverse Transcriptase (Life Technologies, 18080). For each RT, triplicate PCR reactions (20 μL, 10 ng cDNA) were performed using GoTaq qPCR Master Mix (Promega, A6001) to yield six Ct values. Equipment used was the 7500 Real-Time PCR System (Applied Biosystems). These values were converted to the relative amount of RNA by multiplying the fold increase in product per cycle—equivalent to amplification efficiency, to the power of ∆Ct. Amplification efficiencies for each transcript were pre-determined in standard curves. Each value was normalized to the mean obtained for the *Rsp7* (ribosomal protein S7) housekeeper RNA. Primers, 5′-3′: *H3f3a*, GCAG CTAT TGGT GCTT TGC (JRM-1227), ATGT TTCC CCTC ATAG TGGA CTC (JRM-1228), amplicon 173 bp; *H3f3b*, GGAG ATCG CCCA GGAT TTC (JRM-1231), ATGC CATA AAAA CCGC TTCA AC (JRM-1232), amplicon 215 bp; *Rps7*, TGGT CTTC ATTG CTCA GAGG AGG (JRM-609), TGCC ATCC AGTT TCAC ACGG (JRM-610), amplicon 177 bp.

### Immunoblotting

Immunoblotting was performed as described [48]. Tissues were homogenized by trituration with a 19 G needle and syringe in RIPA buffer. Primary Abs used, dilution factor: anti-H3.3, rabbit pcAb, 1000× (Millipore, 09–838); anti-H3 (panH3), rabbit pcAb, 2000× (Abcam, 1791); anti-TUBB (β-tubulin), rabbit mcAb, 1000× (Cell Signaling, 2128); anti-GAPD (glyceraldehyde-3-phosphate dehydrogenase), rabbit mcAb, 1000× (Cell Signaling, 5174). Secondary Abs used, dilution factor: anti-rabbit IgG, goat, horseradish peroxidase conjugate, 15000× (Millipore, 12–348).

### Immunofluorescence in zygotes

The light cycle in the mouse colony was 0630–1830 h. Zygotes were collected at ~1200 h on the day of obtaining a vaginal plug, when usually they were at the mid-pronuclear stage. They were cultured for ~2 h for pronuclei to become more prominent before fixation. For H3S10ph immunofluorescence, WT zygotes were not fixed until at least two in the batch had undergone pronuclear envelope breakdown, indicating entry into mitosis. Immunofluorescence was performed as described [30], except goat serum was used in blocking, and fixation and permeabilization solutions contained 0.2% (w/v) and 0.1% (w/v) BSA, respectively, to prevent zygote sticking. Fixation, permeabilization, washing and secondary Ab incubation steps were performed in volumes of 0.1–0.15 mL in 12-well staining dishes (ProSciTech, H421–12), while blocking and primary Ab incubations were carried out in 10 μL drops under paraffin oil. In mounting, zygotes were transferred to a 1 μL drop of Fluorescence Mounting Medium (Dako, S3023) on a SuperFrost slide, coverslipped, and sealed with nail polish. 5-ethynyl-2′-deoxyuridine (Edu) incorporation and detection was carried out using a Click-iT Edu Imaging Kit (Life Technologies, C10339): freshly isolated zygotes with pronuclei were cultured in 20 μM Edu in medium KSOM-GAA [49] (2 h) prior to fixation. H3K9me3 primary and secondary Ab incubations were carried out immediately before Edu detection. Primary Abs, dilution factor: anti-H3K9me3, rabbit pcAb, 1000× (Abcam, 8898); anti-H3S10ph, rabbit mcAb, 1000× (Cell Signaling, 3377). Secondary Ab, dilution factor: anti-rabbit IgG, goat, 1500×, AlexaFluor 488 (Life Technologies, A-11008). The third wash after secondary Ab staining (10 min, RT) contained DAPI at 1 μg/mL, then zygotes were rinsed in DAPI-free wash solution (1 min) before mounting.

### Genotyping

Tail tips were digested in 0.1 mL of 50 mM Tris-HCl pH 8.0, 0.5% (v/v) Triton X-100, 20 mM EDTA, 400 μg/mL proteinase K, (55°C, overnight). An aliquot was diluted 20× in water, then heat inactivated (70°C, 10 min). This template solution was diluted 10× in the final PCR reaction, performed using MyTaq Red DNA polymerase (Bioline, 21108). Primers: *H3f3a*, CTGG TTTT GGCT GTTT TATC GCTC GG (WT primer, JRM-1272), GCTA TTGC TTTA TTTG TAAC CATT ATAA GCTG C (mutant/conditional, JRM-1461), and AGGG CGCA CTCT TGCG AGC (common, JRM-1276); triplex reaction, amplicons WT—362, and mutant/conditional—205 bp. *H3f3b*, CTCA CCGC TACA GGTA GGC (WT primer, JRM-1465), GCTA TTGC TTTA TTTG TAAC CATT ATAA GCTG C (mutant/conditional, JRM-1461), TCTC CCTC ACCA ATCT CTGG (common, JRM-1466); triplex reaction, amplicons WT—211, mutant/conditional—357. X^*cre*^, specific for the cre cds, TGCT GTTT CACT GGTT ATGC GGCG (JRM-391), TGCC TTCT CTAC ACCT GCGG TGCT (JRM-392); amplicon 304 bp. Y chromosome, nos. 211 and 212 [50].

### Ethics statement

This work was approved by the Animal Ethics Committee of the Murdoch Children’s Research Institute, approval no. A692.

## Supporting Information

S1 Fig Histological evaluation of late gestation *H3f3b* mutant fetuses. Four fetuses were evaluated at 18½ dpc, two days before birth: Uterus-mates no. 1, ♂, *H3f3b* ^+/-^and no. 2, ♂, *H3f3b* ^-/-^. Also uterus-mates no. 3, ♀, *H3f3b* ^+/-^; no. 4, ♂, *H3f3b* ^-/-^. A description of the phenotype of the two mutants is as follows:
no. 2, ♂, *H3f3b* ^-/-^
There were no overt external lesions or gross abnormalities. Crown-rump length (no. 2, ♂, 21 mm; no. 1, ♂, 20 mm) is within expected range for 18½ dpc. The expected crown-rump length of a WT fetus at 18½ dpc is ~23mm [51]. There was no difference observed in the histology compared with the littermate control: Multiple sections demonstrate typical developing nasal region with nasal cartilage septum, cavity and turbinates, oral cavity, larynx, primordium of body of mandible with tongue, developing incisors/molars and follicles of vibrissae. The brain appears to be appropriately differentiated for this age with typical lamination and distinct developing regions. Sections show developing olfactory lobes. The brain cortex is differentiating with neopallial cortex and ventricular zones including surrounding germinal regions. Typical developing midbrain and cerebellar primordium with an external granular layer, Purkinje cell region and developing fissures identified. Also identified is the medulla oblongata, and rostral part of the cervical cord. The development of the pineal gland and the pituitary gland is progressing with increased evidence of differentiation. The development of the eye appears unremarkable with an immature pigmented retinal layer, intra-retinal space (largely an artefact of fixation), retinal nuclear layers (inner and outer), hyaloid cavity, lens (and discernable fibres) and surface epithelium of cornea. The eyelids are fused. Sections through the ear demonstrate unremarkable immature vestibular apparatus including semicircular ducts and discernable crista ampullaris, developing cochlea and a well defined cartilaginous capsule. Multiple sections of the fetus demonstrated all of the major organs. In the thoracic cavity the thymus was prominent with no clear boundaries between the cortex and medulla, a common feature at this stage and typical mitotic figures of lymphoblasts were identified). The heart was present with formed atria/ventricles, valves and unremarkable myocardium. The lungs have well developed lobes with branching bronchi, complex vasculature, alveolar sacs and sac-like primitive alveoli lined by cuboidal epithelium. The cartilaginous skeleton of the trachea is well formed. Unremarkable diaphragm and in the abdominal region, active liver haematopoiesis and megakaryocytes are prominent. The pancreas consists of a secretory cell component and the pancreatic islands are well differentiated. Unremarkable fore and glandular portions of the stomach. The small intestine demonstrated well formed villi and the large intestine with forming crypts. The gut now has well formed mucosa, submucosa, circular and longitudinal muscle layers and goblet cells are conspicuous. The spleen was identified with active haematopoeisis and discernable lymphocytes. The kidneys appeared differentiated with glomeruli and convoluted tubules and the suprarenals (adrenal glands) show typical morphology with distinct medulla/cortex differentiation. The urothelium and detrusor muscle of the bladder appears normal. The salivary glands are identified with distinct glandular trees. Multiple longitudinal sections of the spinal cord demonstrates white and grey matter (containing neurons and early lamination) with surrounding vertebral body, spinal ganglia, striated muscle and bone marrow. Unremarkable immature testes with distinct seminiferous tubules lined with spermatogonia. Sections shows longitudinal/oblique levels through the hind foot plate with divergent digits and hind leg with typical developing joint, cartilage, areas of ossification, bone marrow, skeletal muscle, brown fat deposits and unremarkable skin with developing dermal appendages. A salient feature of this developmental stage is the appearance of ossification centres in the upper thoracic region of the vertebral column. Ossification centres of the cervical vertebral bodies could not be evaluated due to decapitation artefact. Levels through the placenta show the maternal decidua, spongiotrophoblast layer, characteristic embryo-derived giant cell trophoblast layer, portion of yolk sac, the labyrinth (site of maternal-fetal exchange) and central artery. The labyrinth contains numerous fetal blood vessels (containing anuclear mature fetal blood) and maternal blood sinuses. Typical chorionic villi can be seen along with morphologically normal giant cell trophoblasts and spongioblasts. The placenta does not appear to be disrupted and there is no morphological difference when compared to the control sample.
no. 4, ♂, *H3f3b* ^-/-^
There were no overt external lesions or gross abnormalities. Crown-rump length (no. 3, ♂, 20 mm; no. 4, ♀, 20 mm) is within expected range for 18½ dpc. There was no observable difference in the histology compared with the littermate control: Findings were equivalent to those for no. 2 (above).
(PDF)

S2 Fig TUNEL staining of adult seminiferous tubules. Bars, 25 μm.
(TIF)

S3 Fig Spermatocyte and spermatid purification by flow cytometry. Propidium iodide-negative or live cells were plotted against Hoechst 33342 fluorescence (*y*-axis) versus forward scatter (*x*-axis) signals. Sorted larger 4c cells (pachytene spermatocytes) and smaller 1c cells (round spermatids) are indicated.
(TIF)
