# Supplementary material for: Contribution of the Two Genes Encoding Histone Variant H3.3 to Viability and Fertility in Mice
Source: PLoS Genet. 2015 Feb 12;11(2):e1004964. doi: 10.1371/journal.pgen.1004964 (PMC4335506; doi:10.1371/journal.pgen.1004964)
Supplement: S1 Fig — Four fetuses were evaluated at 18½ dpc, two days before birth: Uterus-mates no. 1, ♂, H3f3b +/-and no. 2, ♂, H3f3b -/-. Also uterus-mates no. 3, ♀, H3f3b +/-; no. 4, ♂, H3f3b -/-. A description of the phenotype of the two mutants is as follows: no. 2, ♂, H3f3b -/- There were no overt external lesions or gross abnormalities. Crown-rump length (no. 2, ♂, 21 mm; no. 1, ♂, 20 mm) is within expected range for 18½ dpc. The expected crown-rump length of a WT fetus at 18½ dpc is ~23mm [51]. There was no difference observed in the histology compared with the littermate control: Multiple sections demonstrate typical developing nasal region with nasal cartilage septum, cavity and turbinates, oral cavity, larynx, primordium of body of mandible with tongue, developing incisors/molars and follicles of vibrissae. The brain appears to be appropriately differentiated for this age with typical lamination and distinct developing regions. Sections show developing olfactory lobes. The brain cortex is differentiating with neopallial cortex and ventricular zones including surrounding germinal regions. Typical developing midbrain and cerebellar primordium with an external granular layer, Purkinje cell region and developing fissures identified. Also identified is the medulla oblongata, and rostral part of the cervical cord. The development of the pineal gland and the pituitary gland is progressing with increased evidence of differentiation. The development of the eye appears unremarkable with an immature pigmented retinal layer, intra-retinal space (largely an artefact of fixation), retinal nuclear layers (inner and outer), hyaloid cavity, lens (and discernable fibres) and surface epithelium of cornea. The eyelids are fused. Sections through the ear demonstrate unremarkable immature vestibular apparatus including semicircular ducts and discernable crista ampullaris, developing cochlea and a well defined cartilaginous capsule. Multiple sections of the fetus demonstrated all of the major orga [file pgen.1004964.s001.pdf]

#1, H3f3b (+/-)
littermate to #2

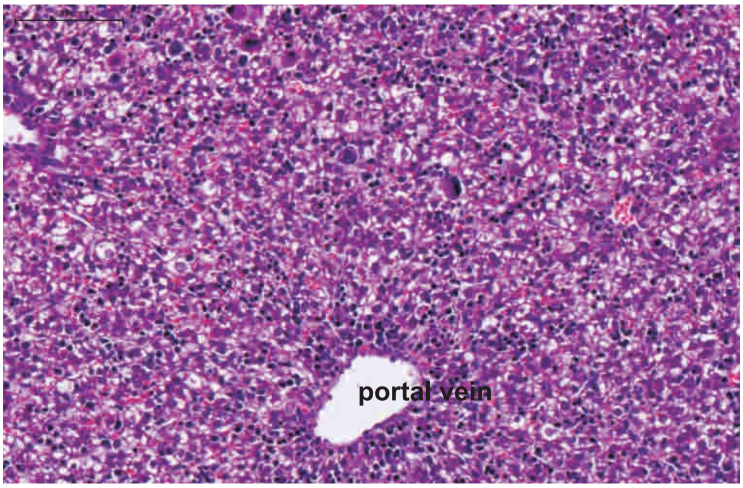

#1 control Liver x20 29612

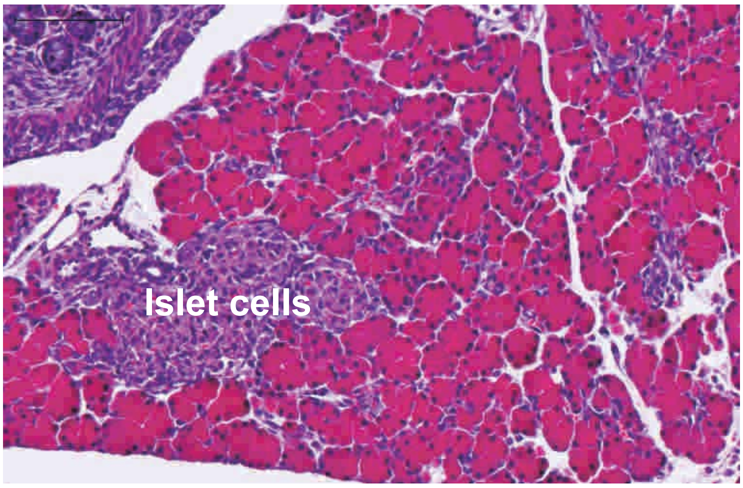

#1 control pancreas x20 29612

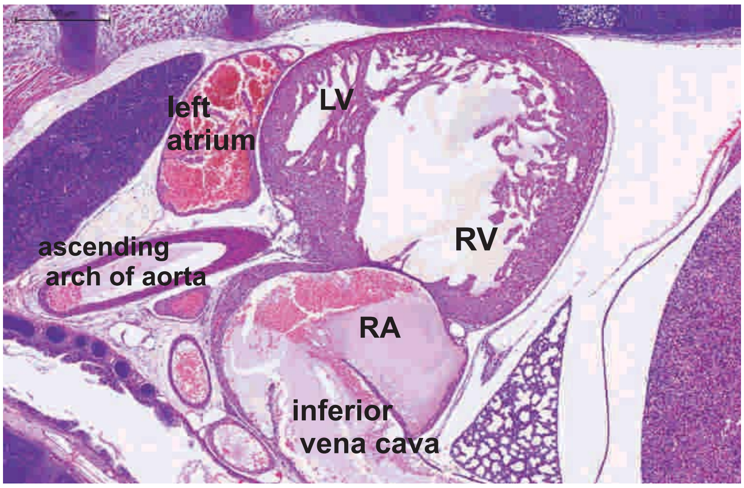

#1 control heart x5 29612

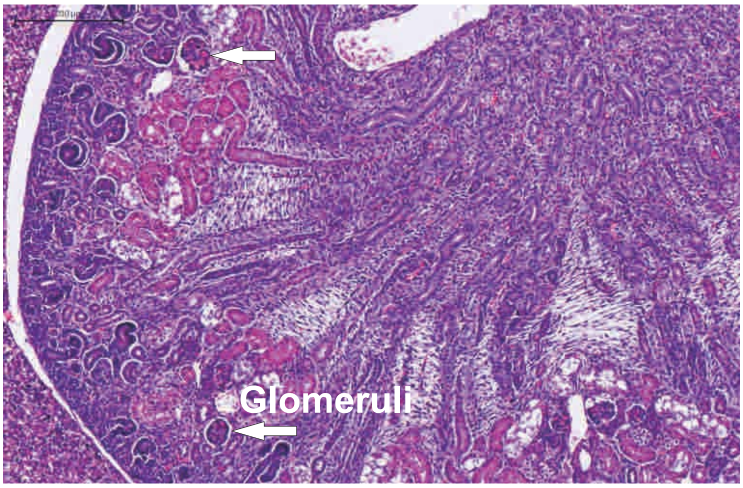

#1 control kidney x10 29612

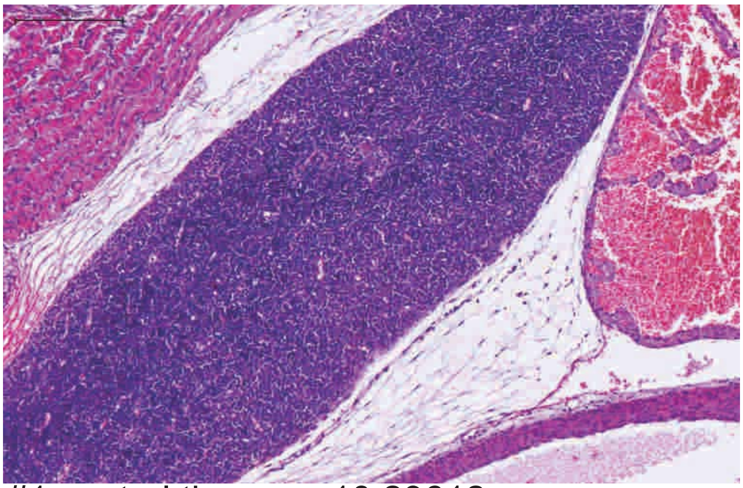

#1 control thymus x10 29612

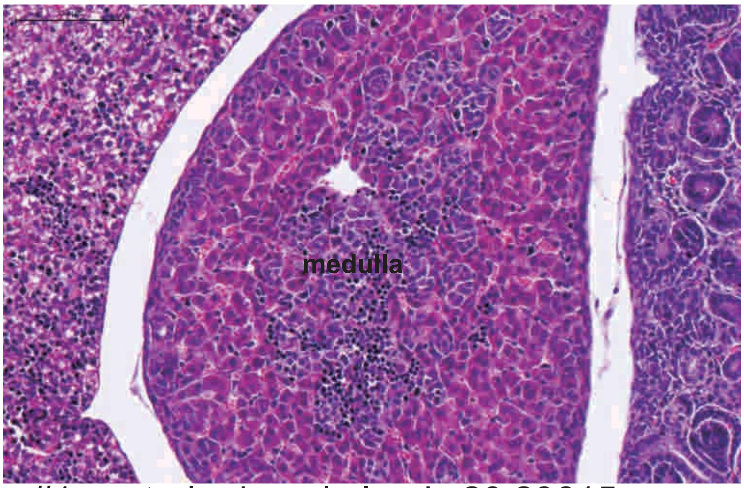

#1 control adrenal gland x20 29615

Figure S1

APN13/052MCRI

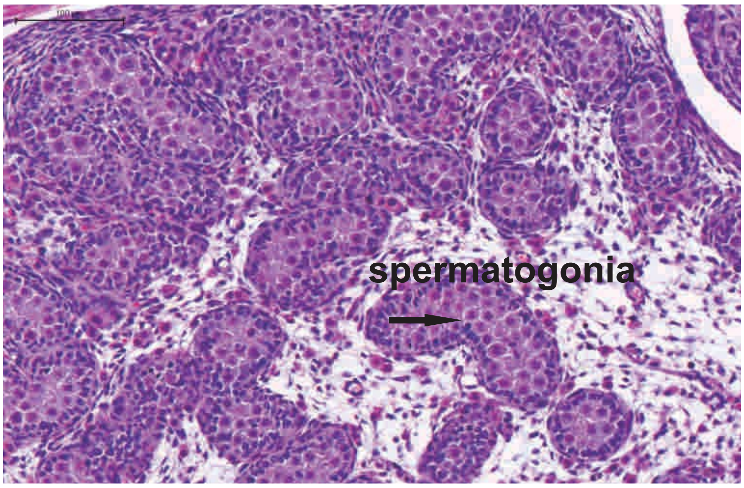

#1 control testis x20 29615

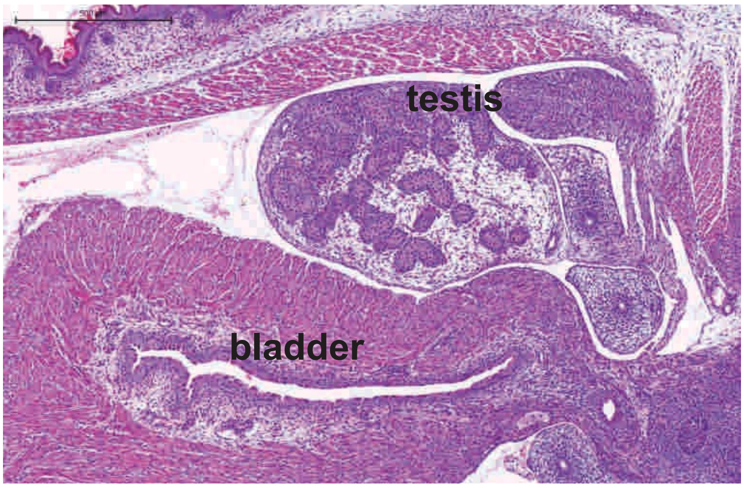

#1 control bladder & testis x5 29615

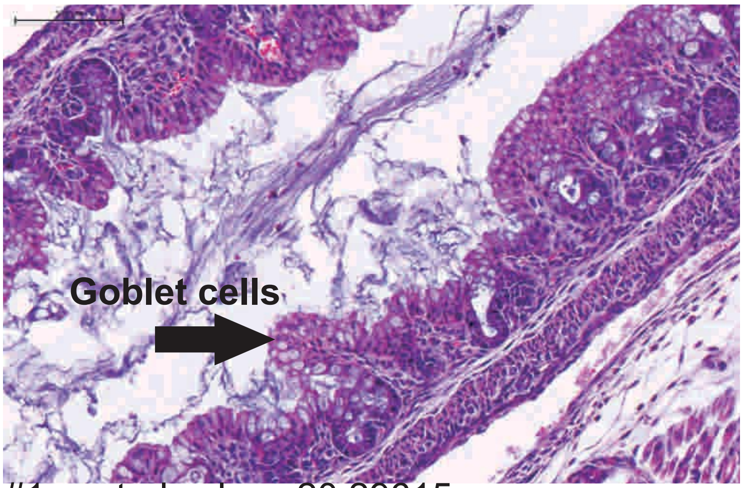

#1 control colon x20 29615

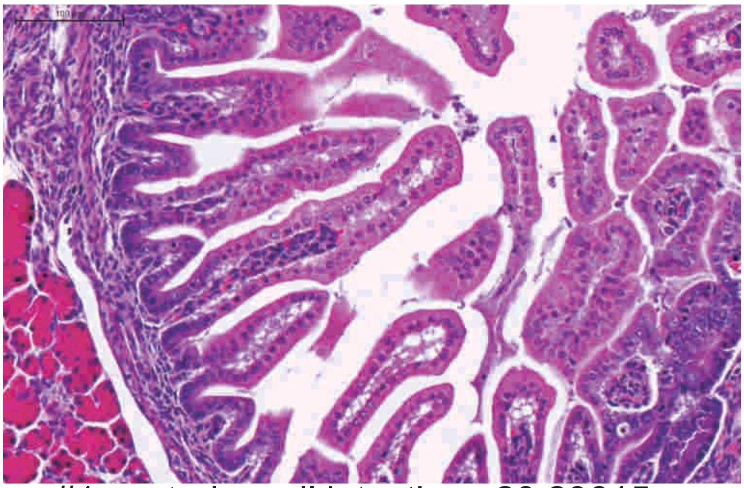
#1 control small intestine x20 29615

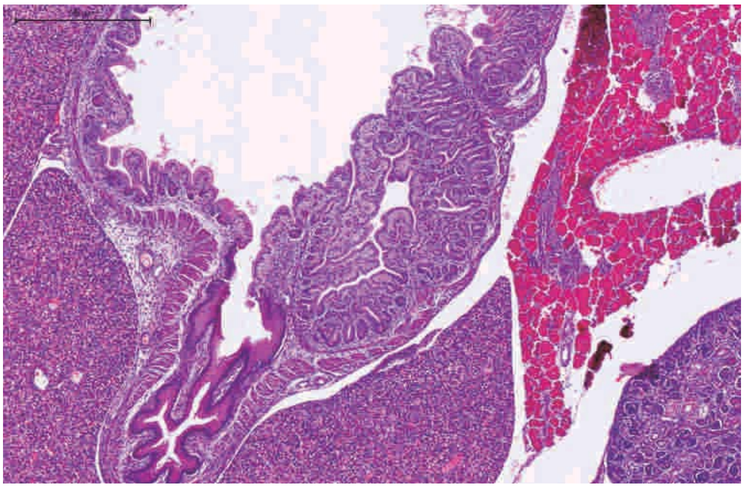
#1 control stomach x5 29624

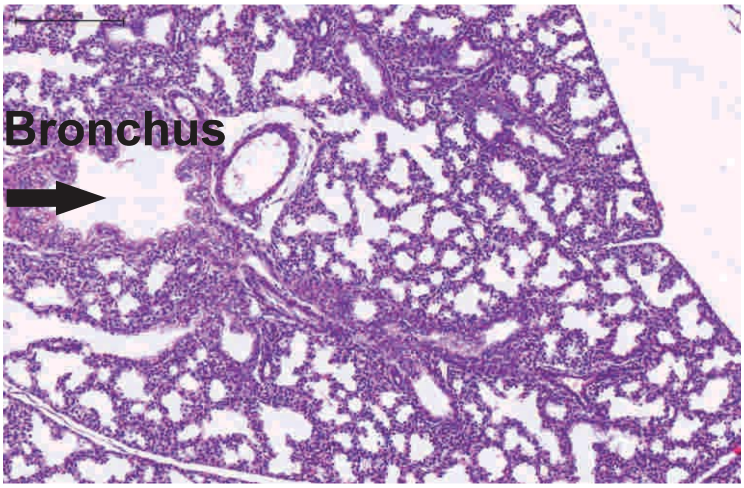

#1 control lung x10 29615

APN13/052MCRI

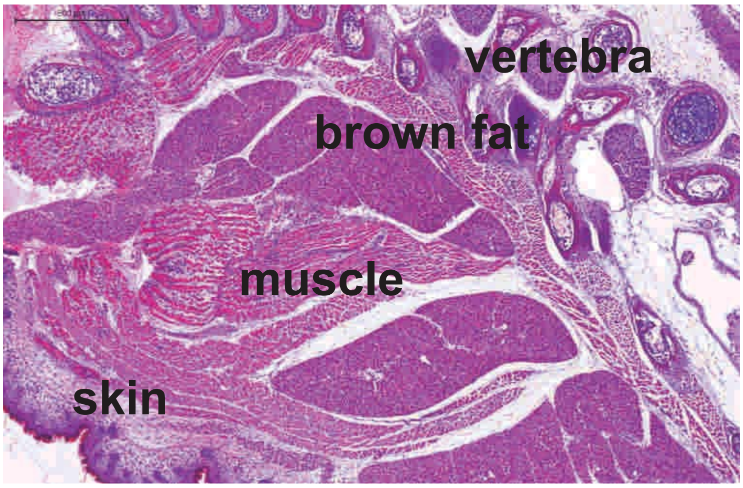

#1 control skin brown fat & vertebra x5 29621

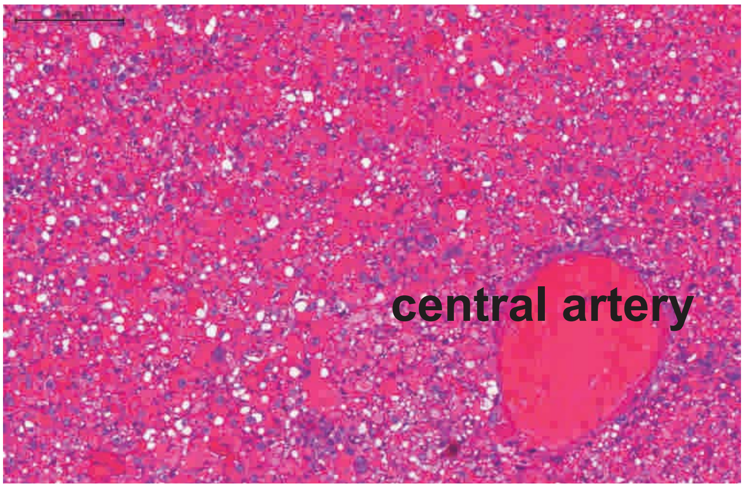

#1 cont placenta-decidua x10 29620

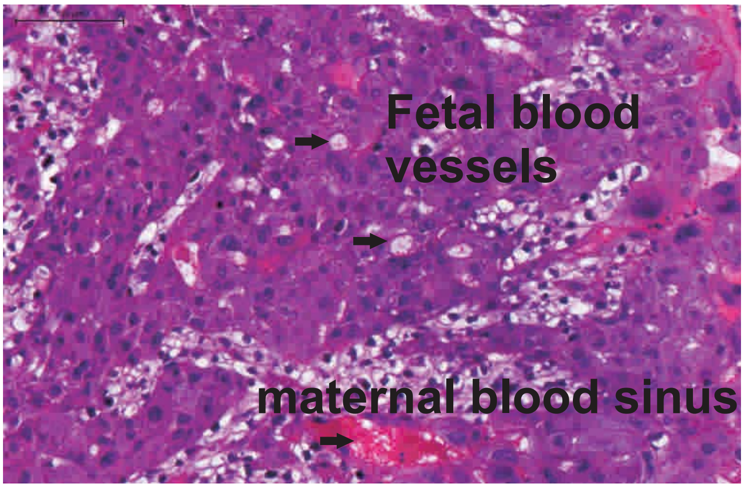

#1 control placenta- labyrinth x20 29614

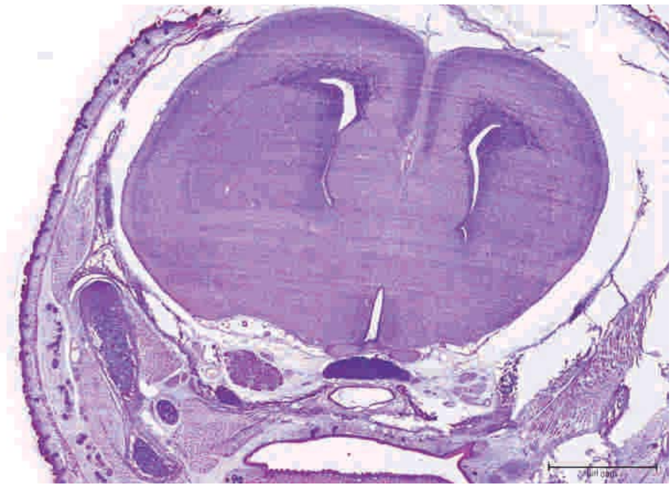

#1 cont forebrain x2 29632

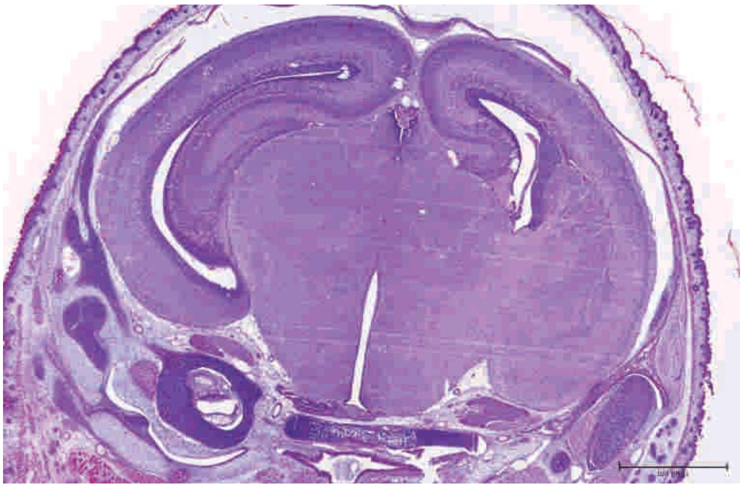

#1 cont mid brain x2 29635
Note: oblique plane

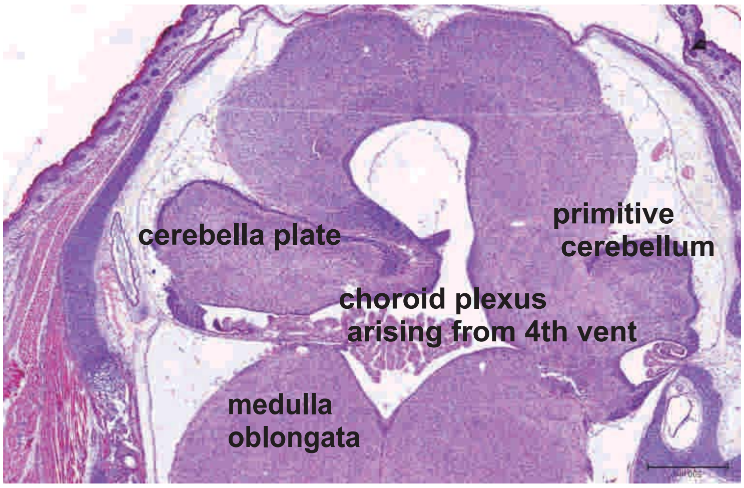

#1 cont developing cerebellum x3 29642

APN13/052MCRI

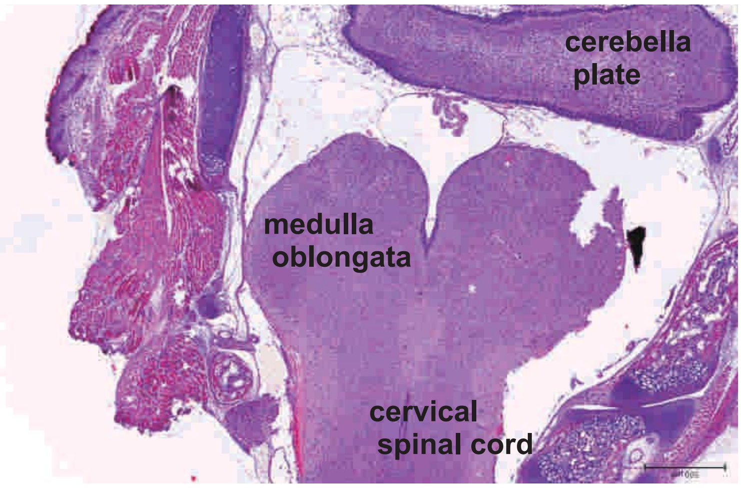

#1 cont hind brain x3 29644

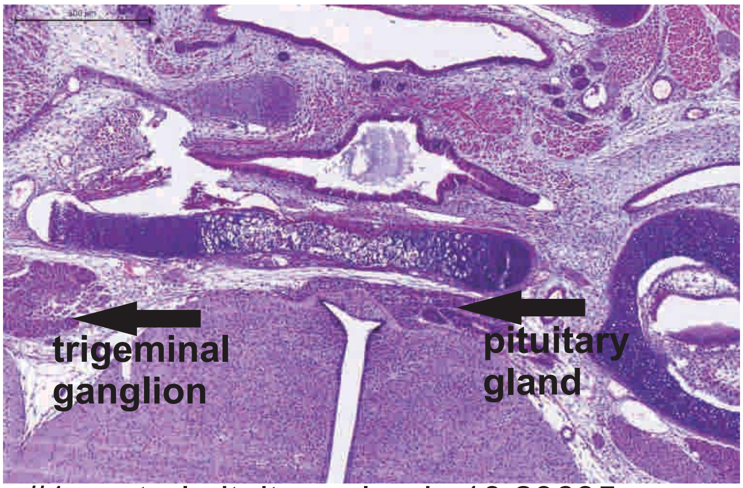

#1 control pituitary gland x10 29635

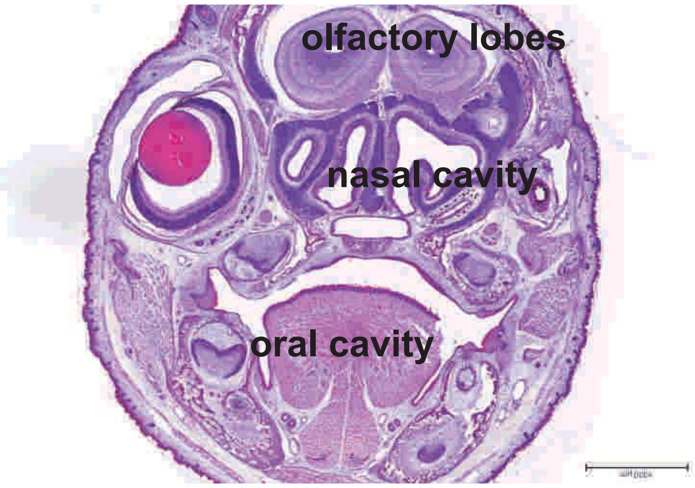

#1olfactory lobes & eye x2 29628

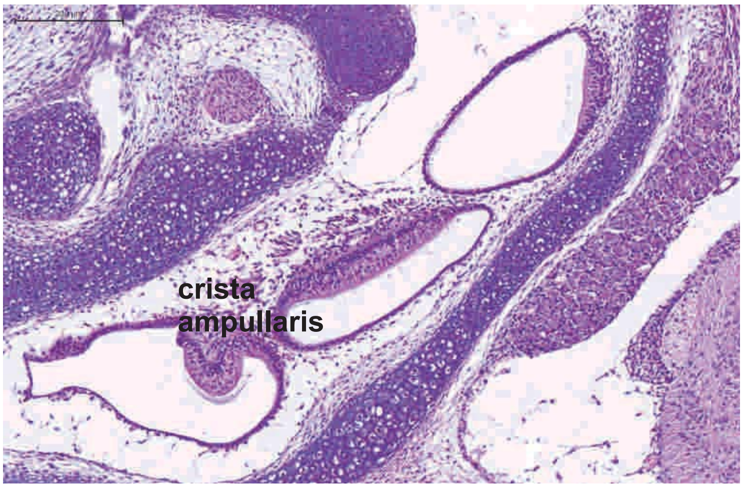

#1 cont semicircular canals
x10 29639

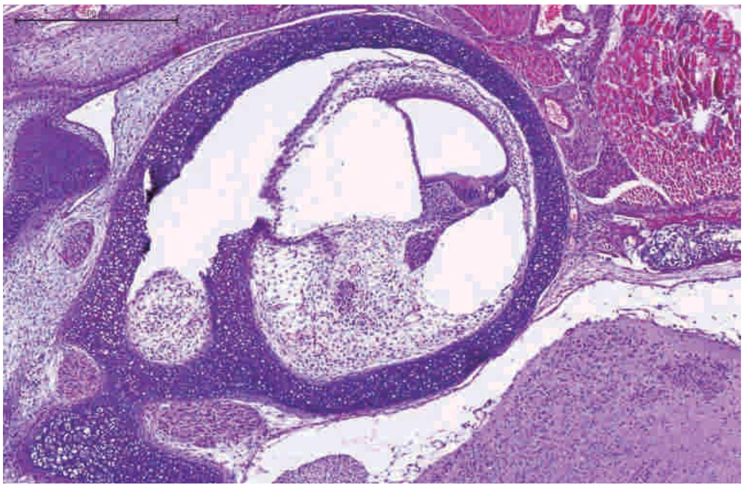

#1cont developing
cochlea x10 29638

APN13/052MCRI

Figure S1
page 5 of 14

#2, H3f3b (-/-)
littermate to #1

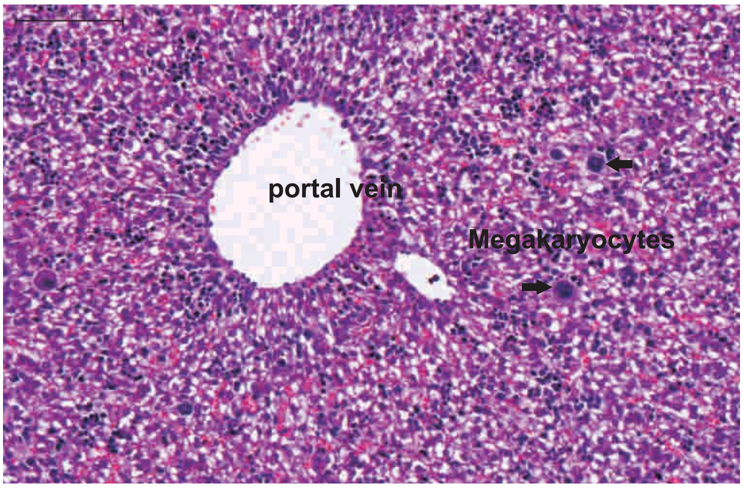

#2 Liver x20 29648

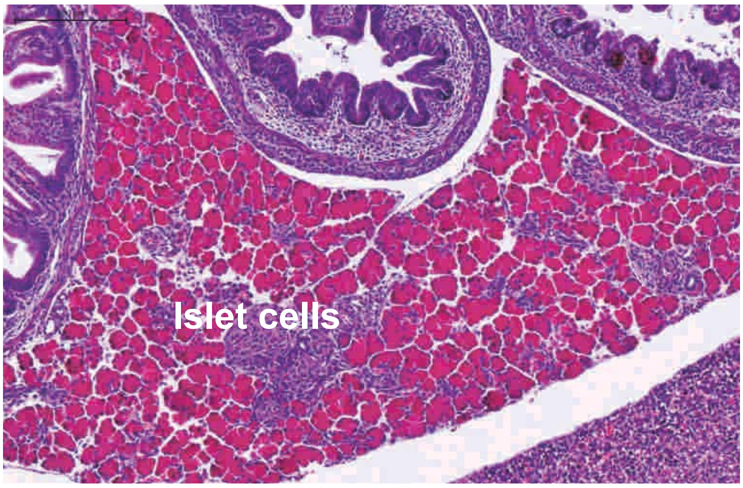

#2 Pancreas x10 29657

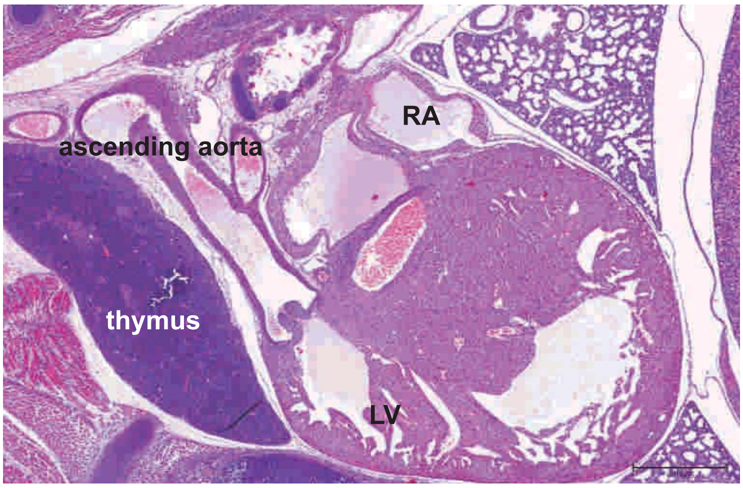

#2 Heart x5 29645

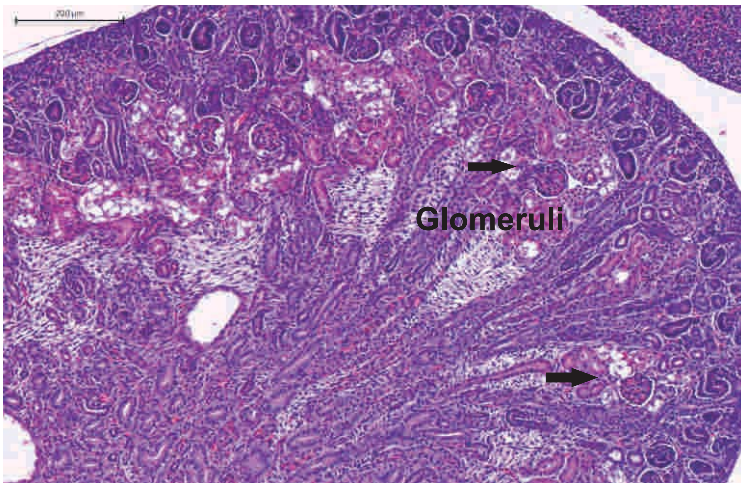

#2 Kidney x10 29648

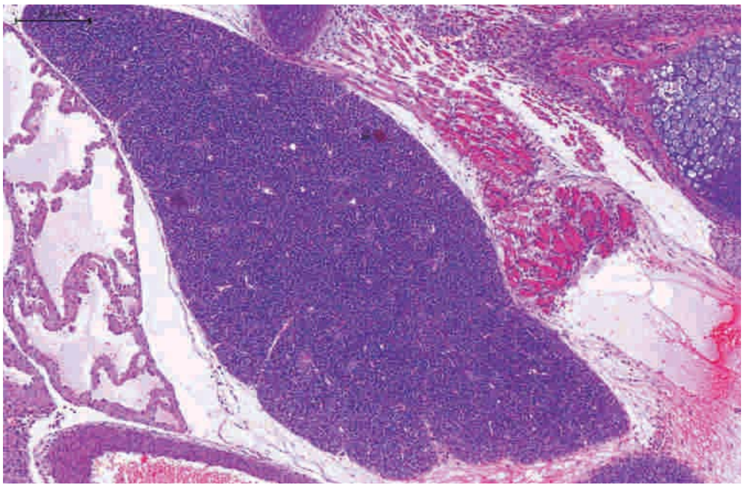

#2 Thymus x5 29649

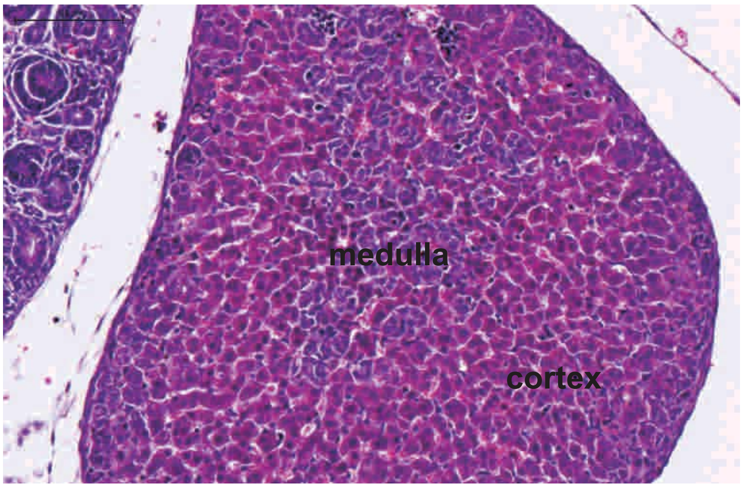

#2 Adrenal gland x10 29647

APN13/052MCRI

Figure S1

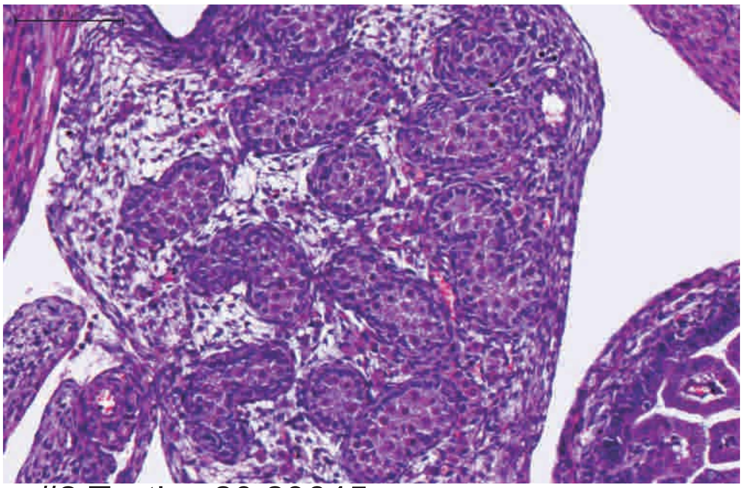

#2 Testis x20 29645

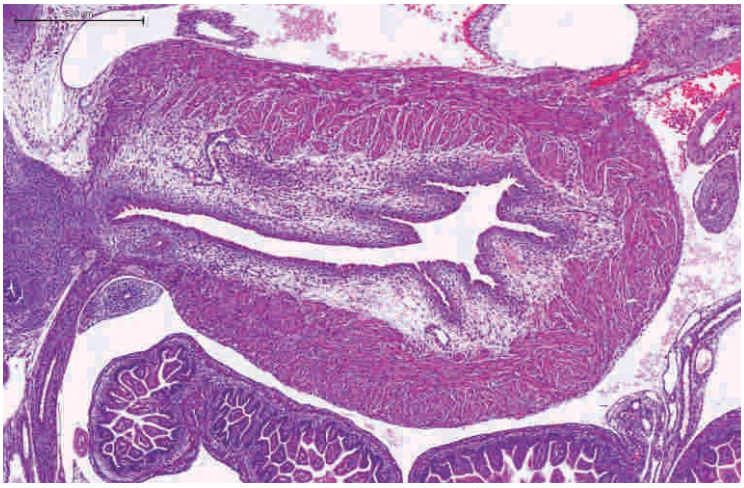

#2 Bladder x10 29648

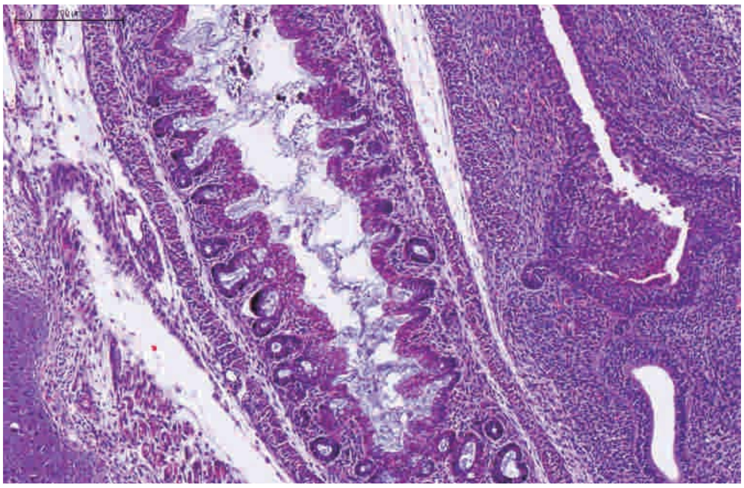

#2 Colon x10 29649

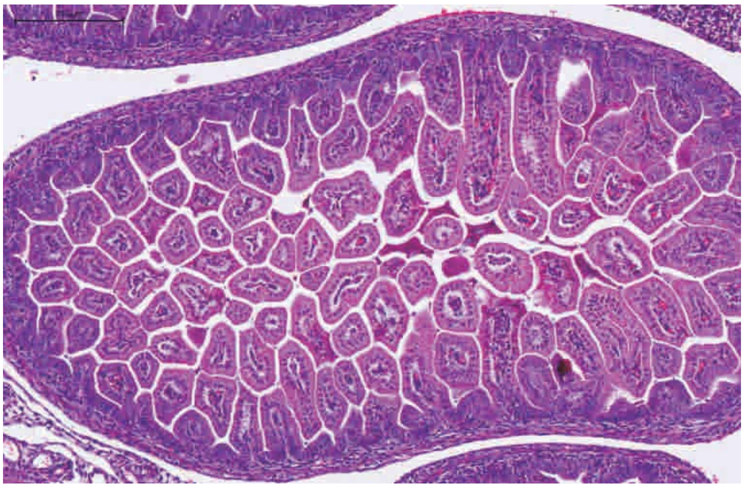

#2 Small intestine x10 29649

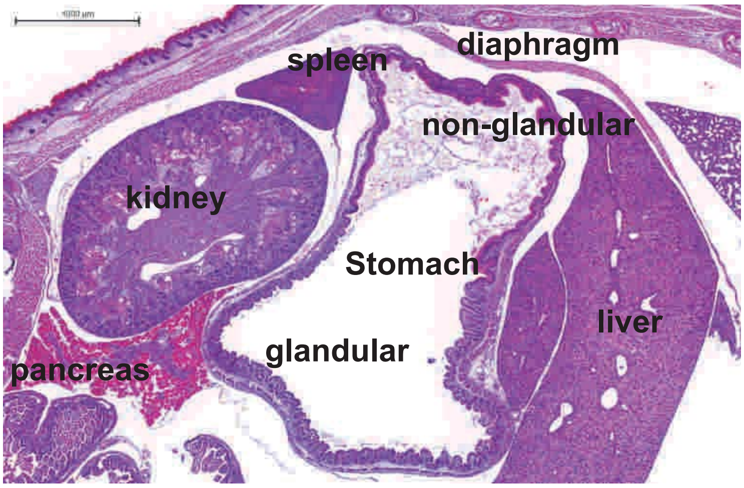

#2 Stomach 29648

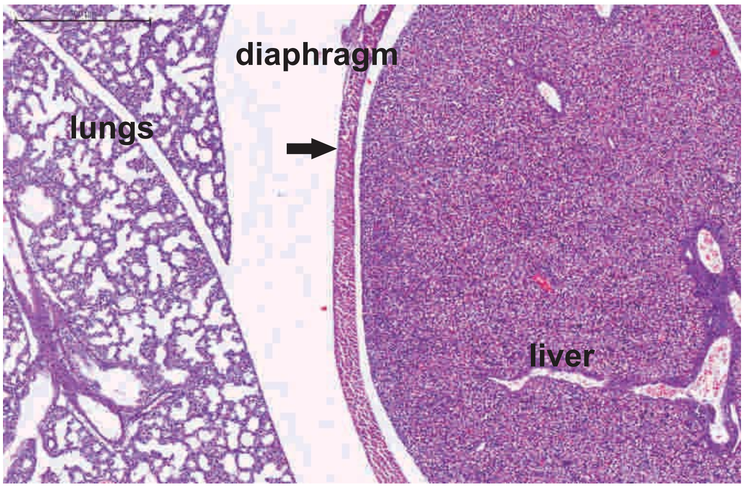

#2 Diaphragm x5 29656

APN13/052MCRI

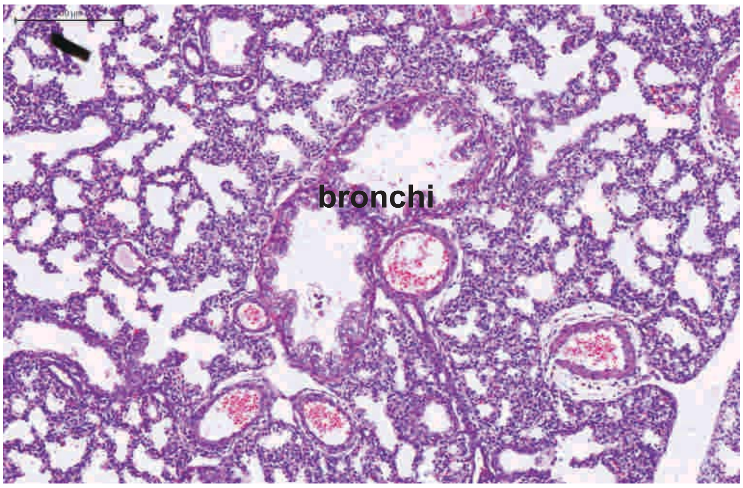

#2 lung x10 29656

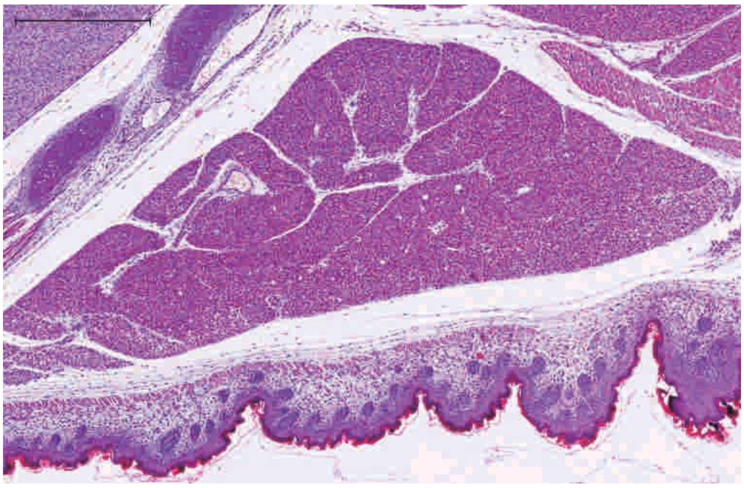

#2 skin and brown fat x5 29649

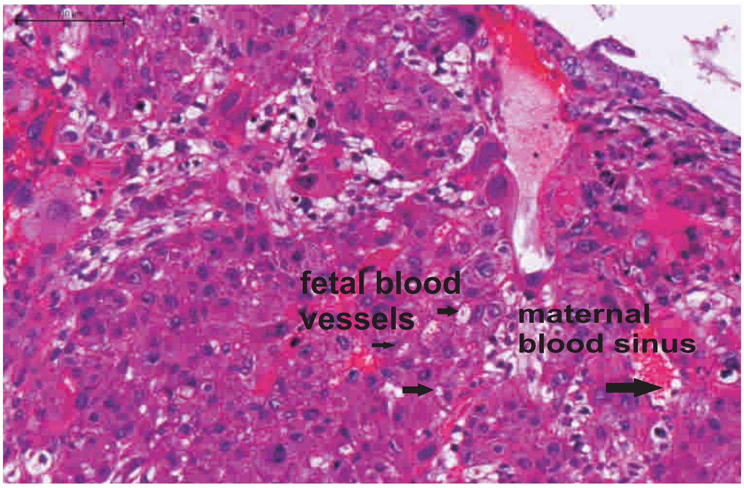

#2 placenta labyrinth x20 29655

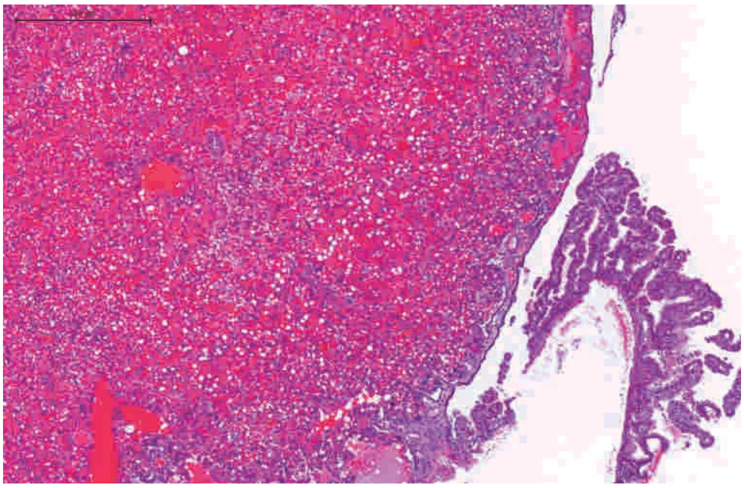

#2 placenta-deciduax5 29648

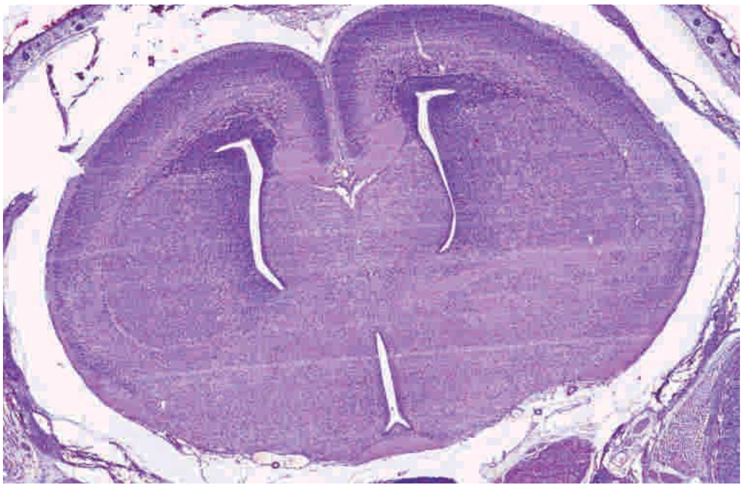

#2 forebrain x3 29667

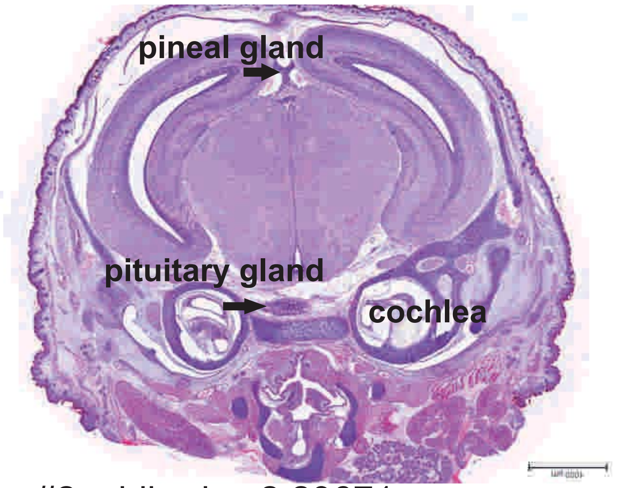

#2 midbrain x2 29671

APN13/052MCRI

Figure S1

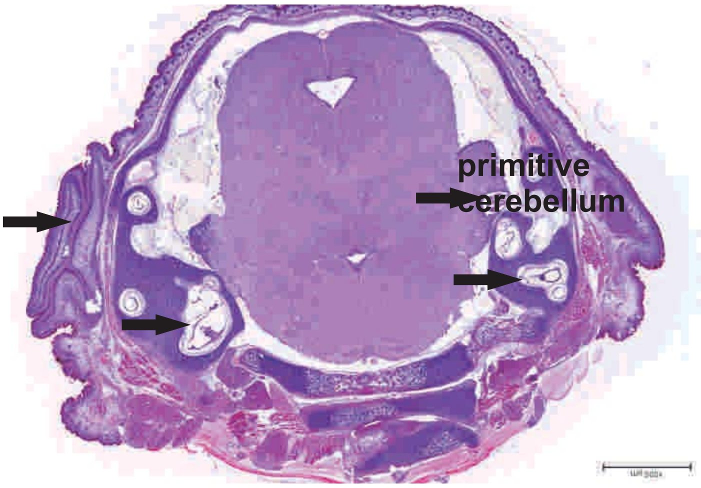

#2 semicircular canal & pina x2 29677

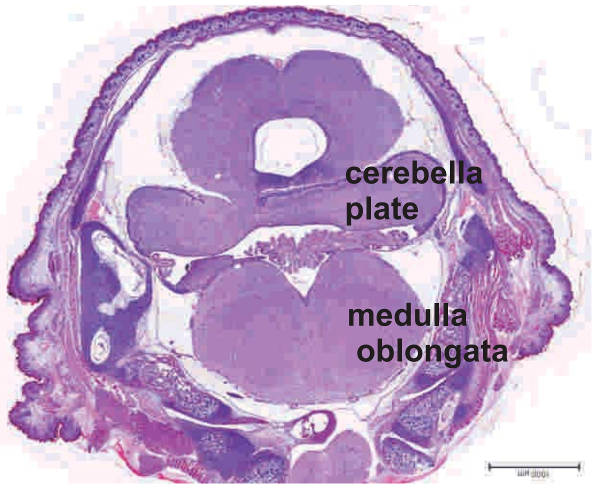

#2 cerebellum x2 29677

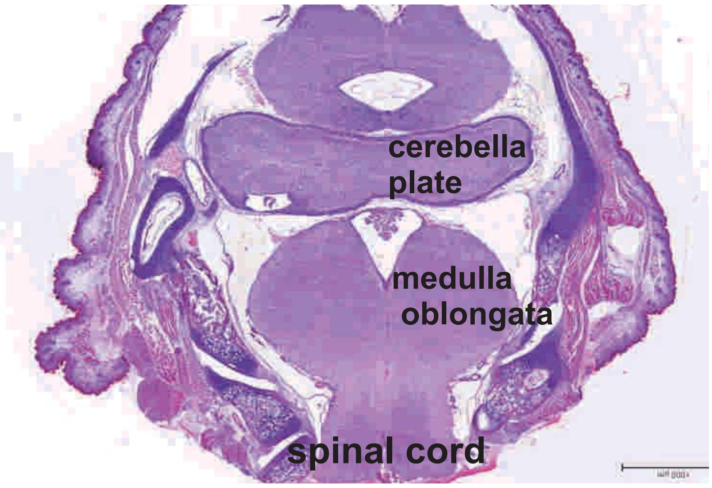

#2 hindbrain x2 29678

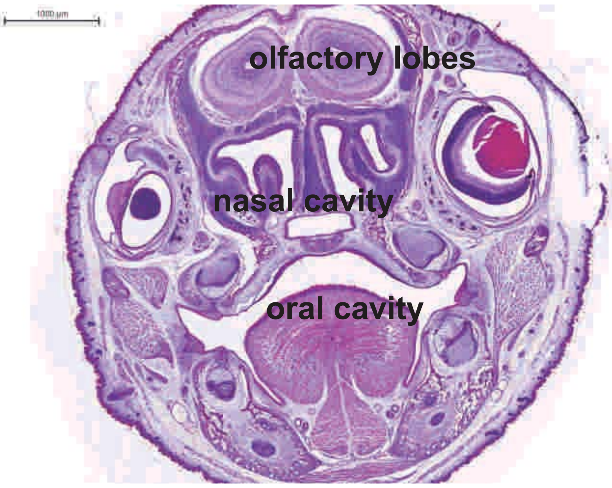

#2 nasal & oral cavity
x2 29663

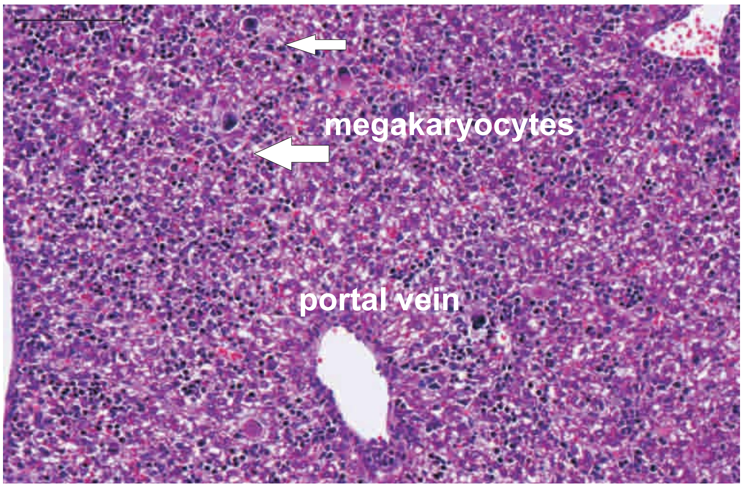

#3 liver x20 29701

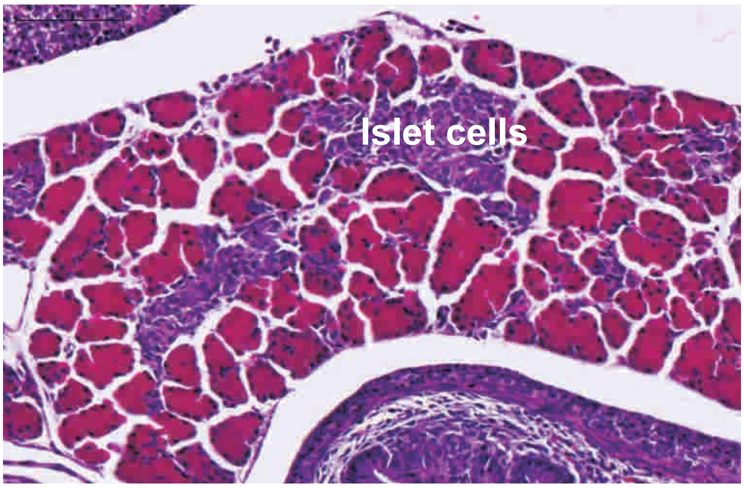

#3 pancreas x20 29685

#3, H3f3b (+/-)
littermate to #4

APN13/052MCRI

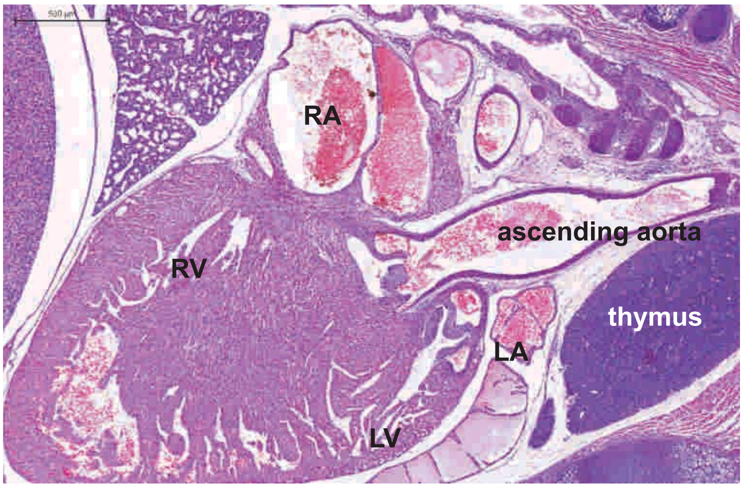

#3 Heart x5 29689

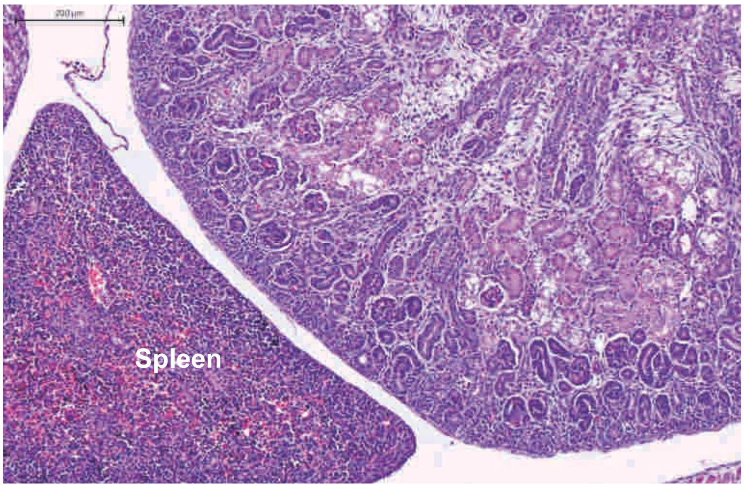

#3 Kidney & Spleen x10 29703

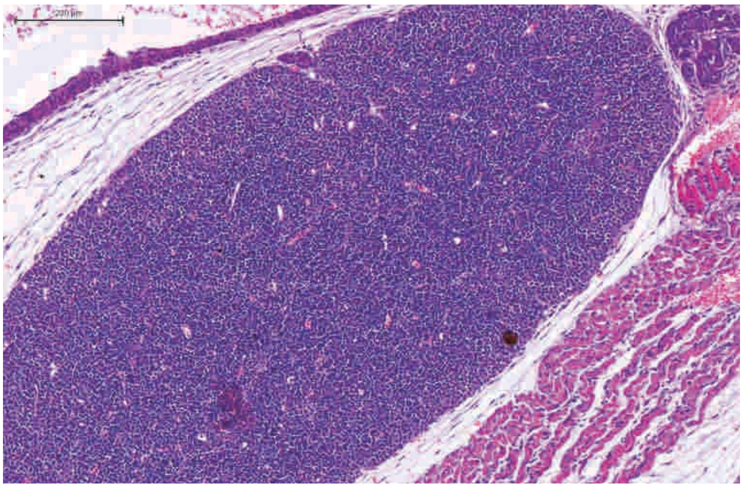
#3 Thymus x10 29687

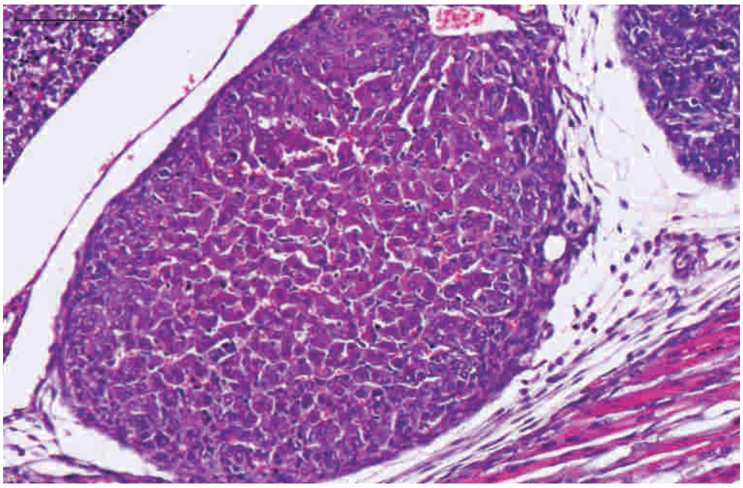
#3 Adrenal gland x20

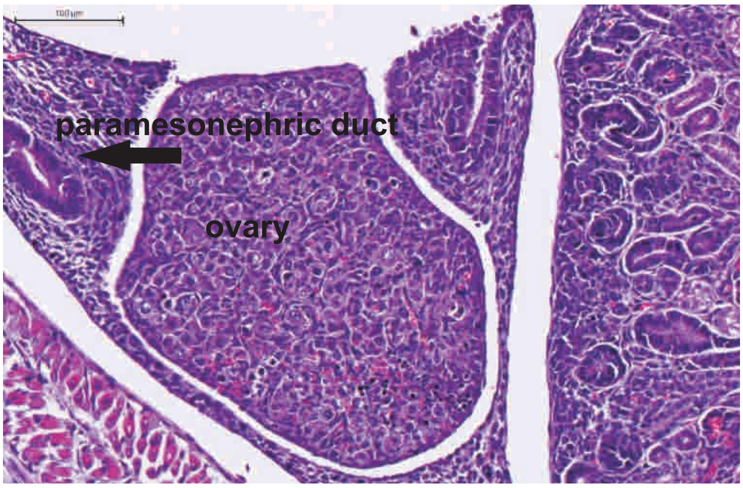

#3 ovary with
typical “dotted” morphology x20 29687

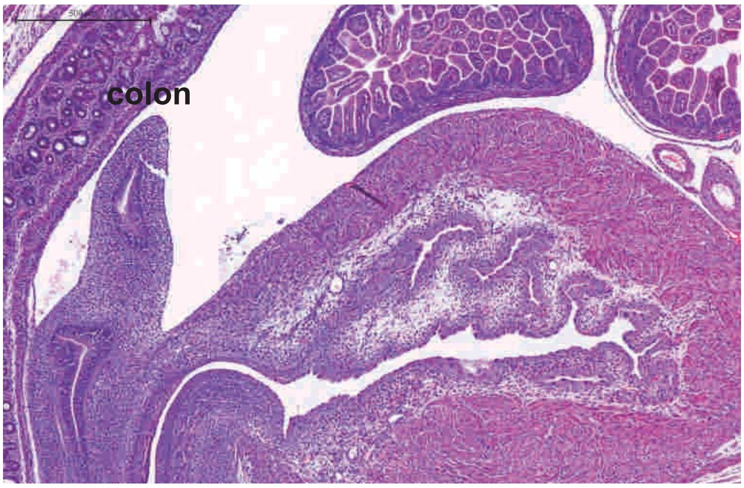

#3 bladder & colon x5 29681

Figure S1

APN13/052MCRI

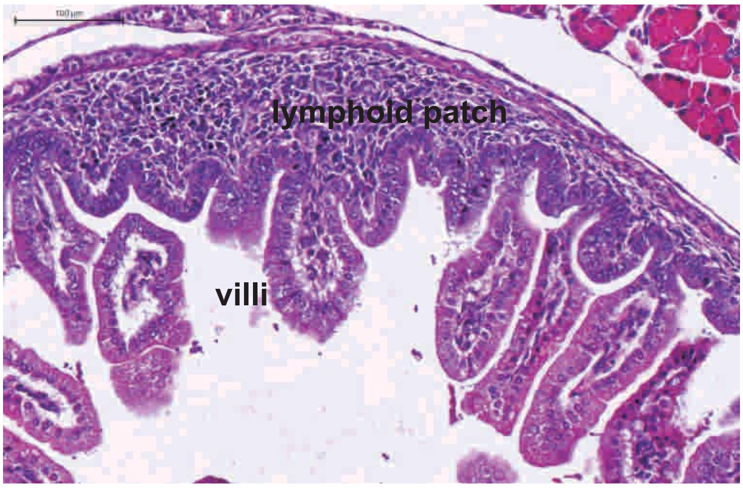

#3 small intestine x20 29693

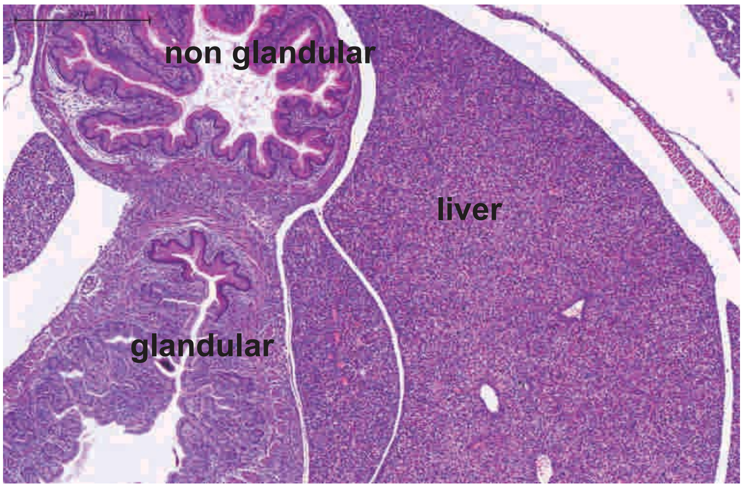

#3 stomach x5 29701

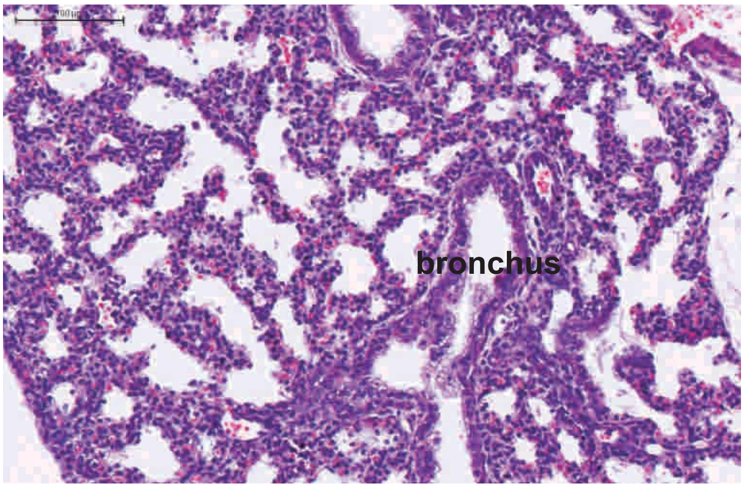

#3 lung x20 29701

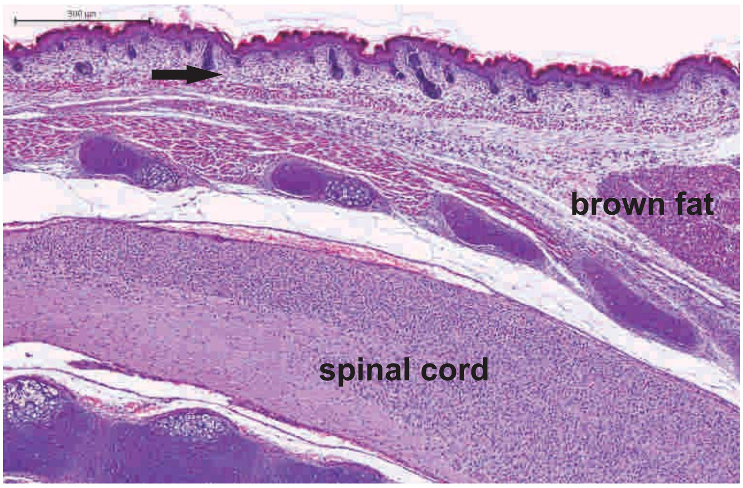

#3 skin & brown fat x5 29693

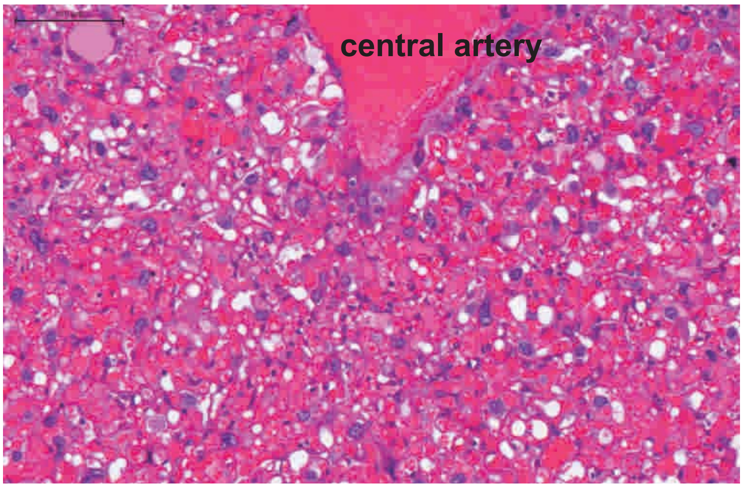

#3 placenta-decidua x20 29701

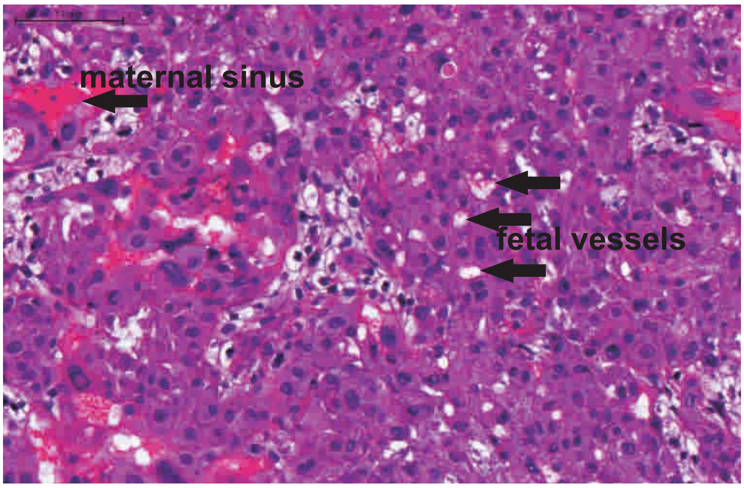

#3 placenta-labyrinth x20 29681

APN13/052MCRI

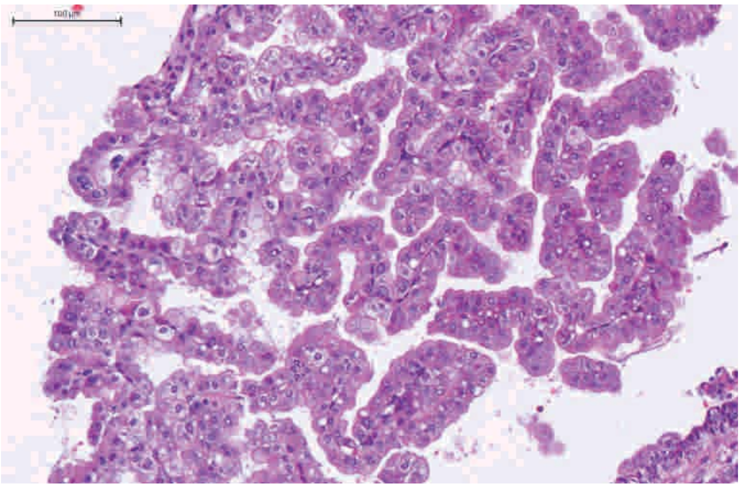

#3 yolk sac x20 29701

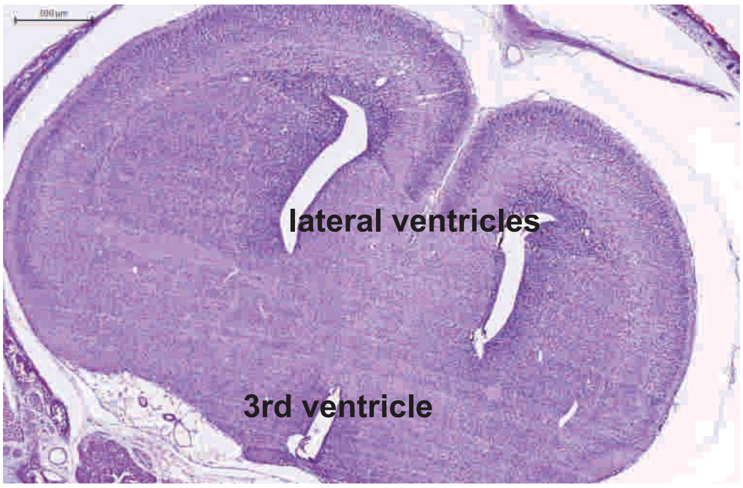

#3 forebrain x3 29696

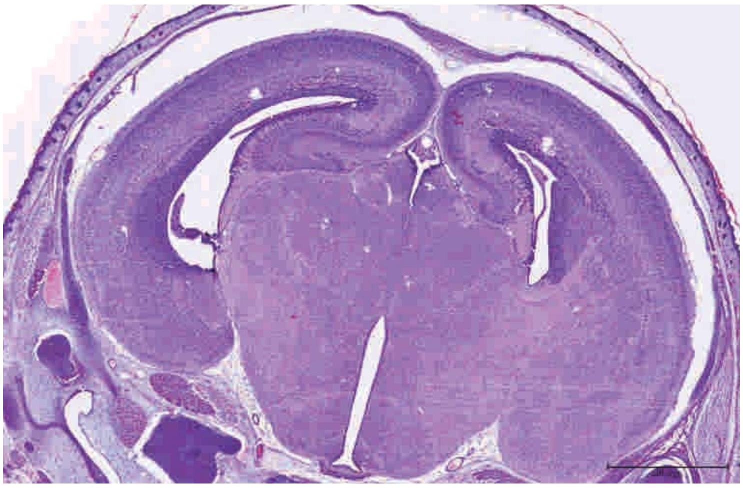

#3 midbrain x2 29702

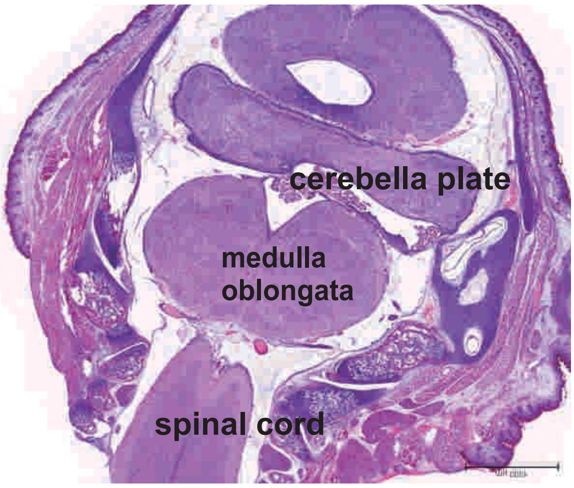

#3 hindbrain x2 29714

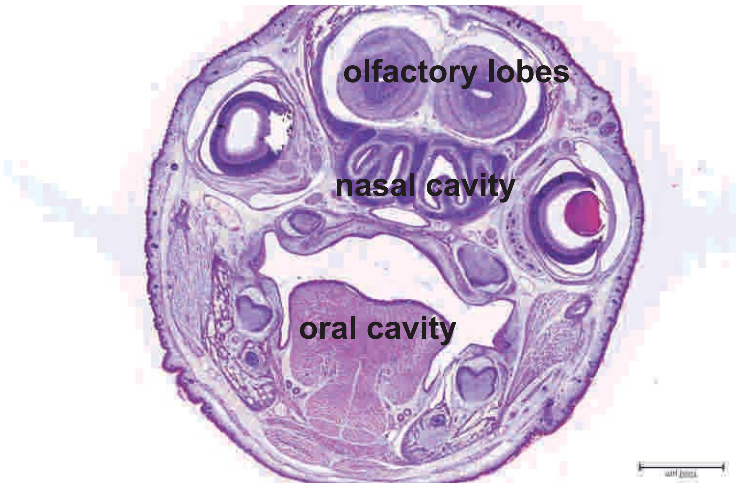

#3 Head(rostral) x2 29690

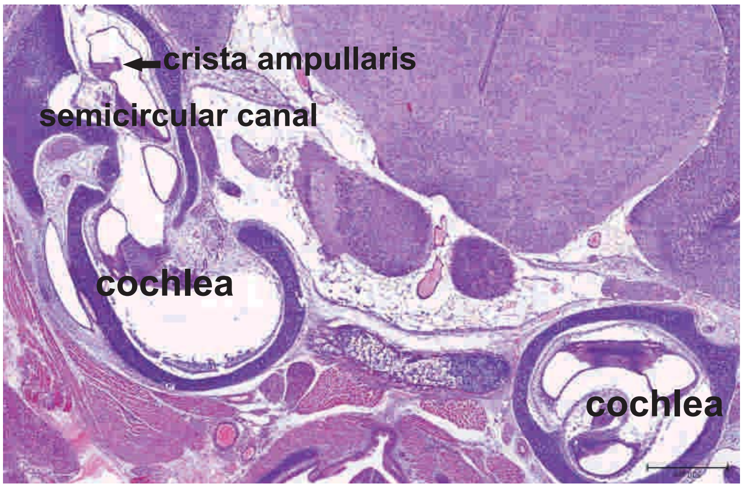

#3 inner ear x3 29706

#4, H3f3b (-/-)
littermate to #3

APN13/052MCRI

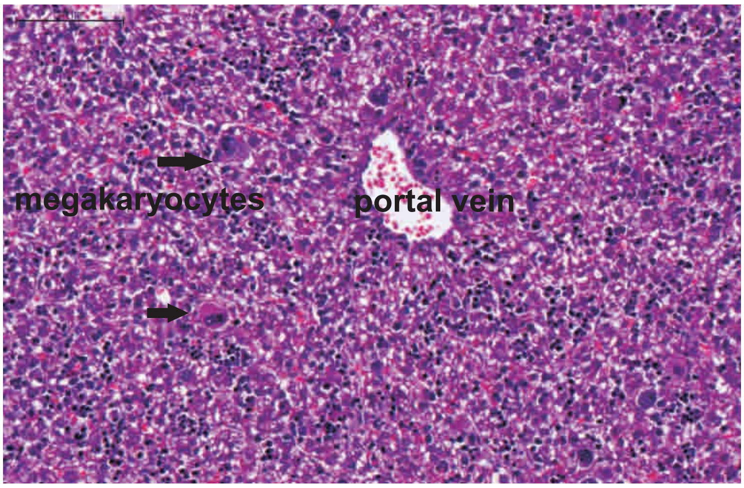

#4 Liver x20 29715

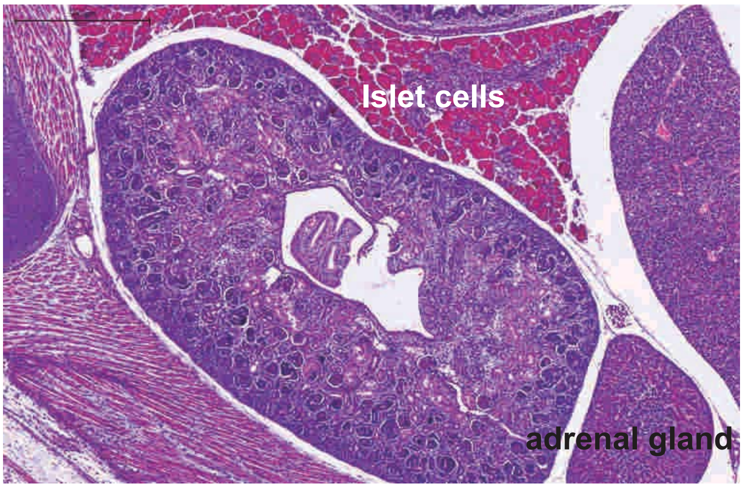

#4 kidney, adrenal gland & pancreas x5 29715

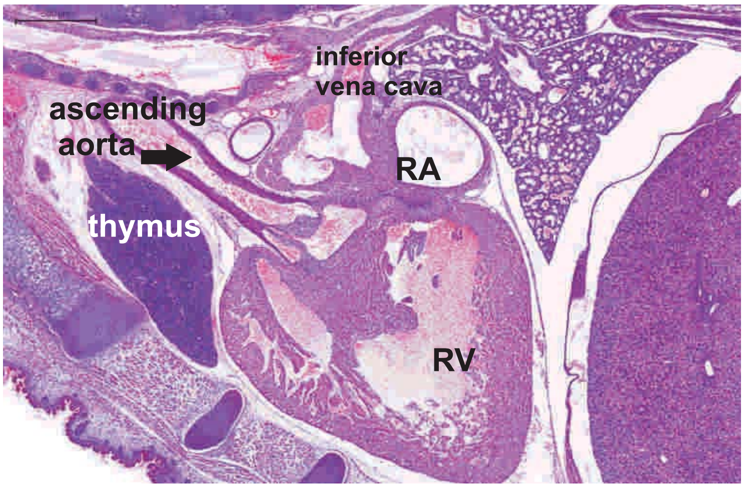

#4 Heart x2 29725

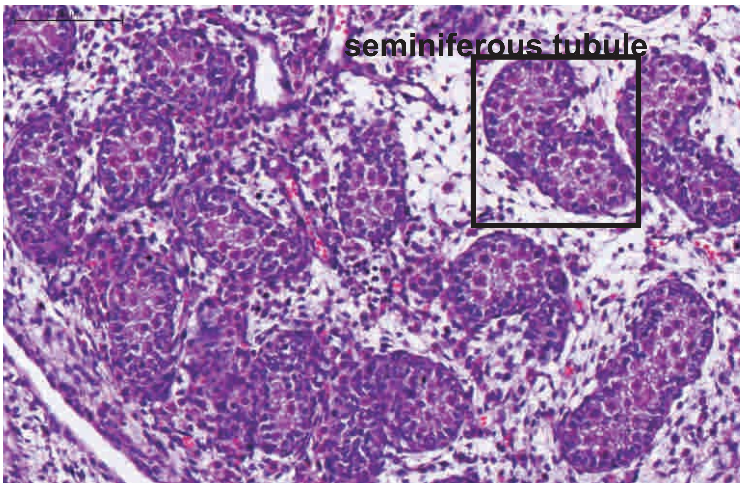

#4 Testis x20 29715

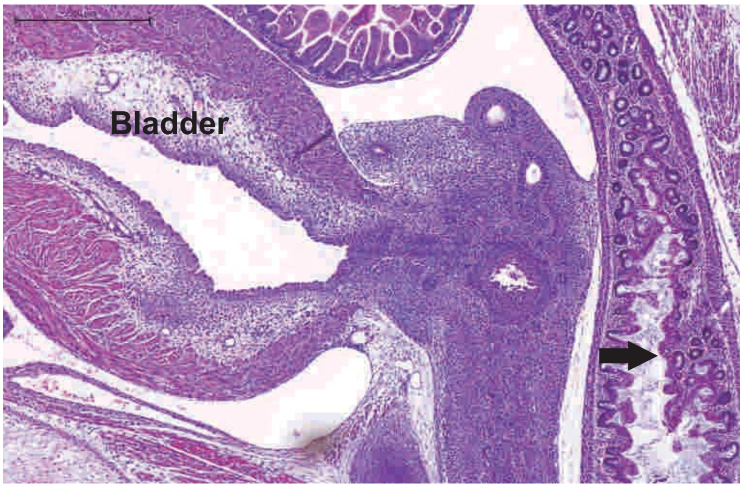

#4 Bladder & colon x5 29725

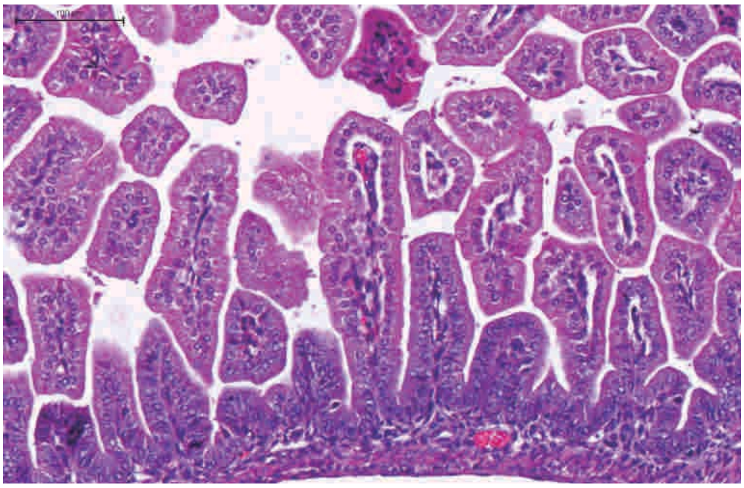

#4 Small intestine x20 29725

APN13/052MCRI

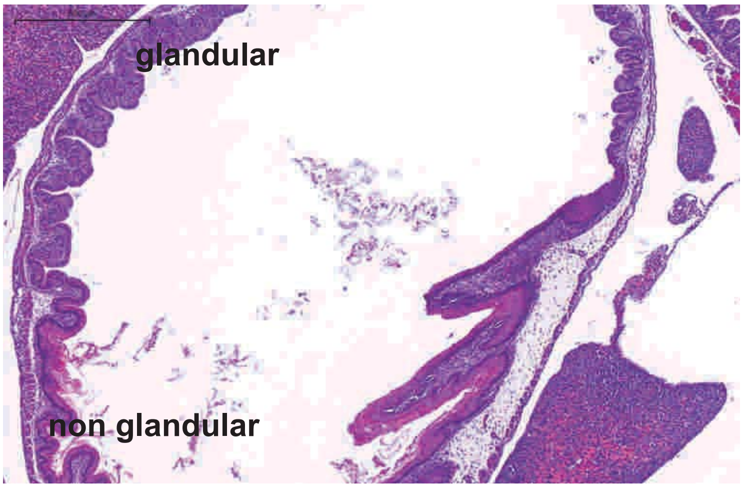

#4 Stomach x5 29725

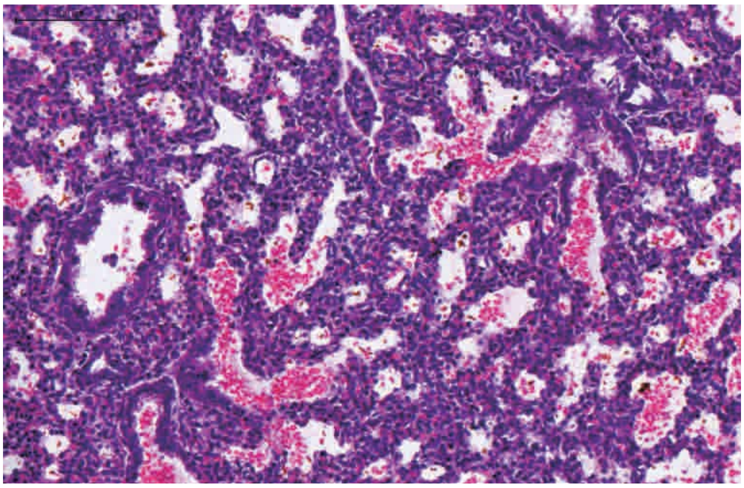

#4 lung x20 29715

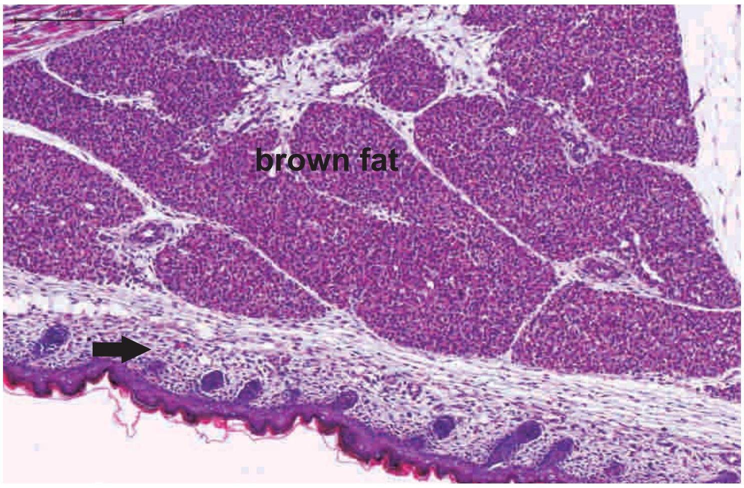

#4 Skin & brown fat x10 29731

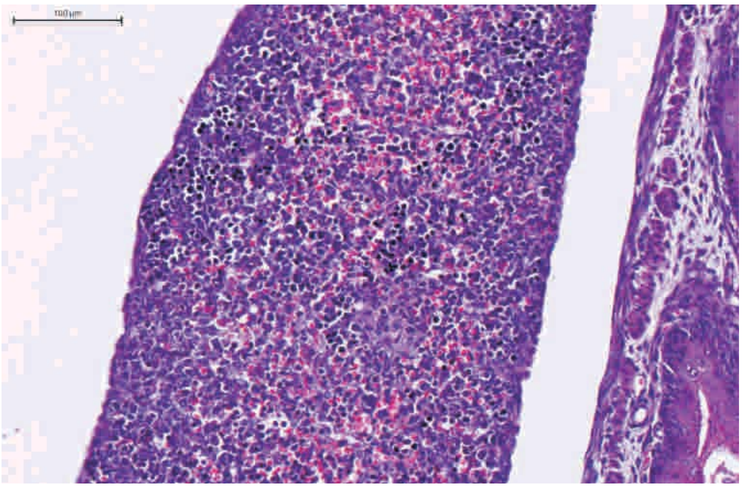

#4 Spleen x20 29731

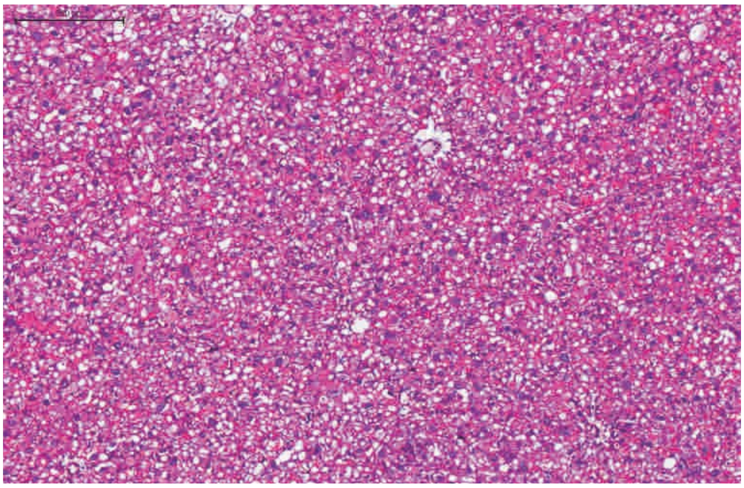

#4 Placenta- decidua x10 29731

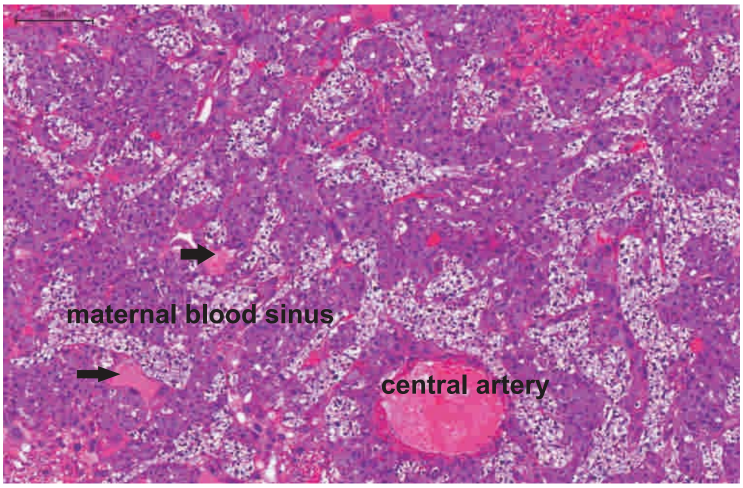

#4 Placenta-labyrinth x7 29725

APN13/052MCRI

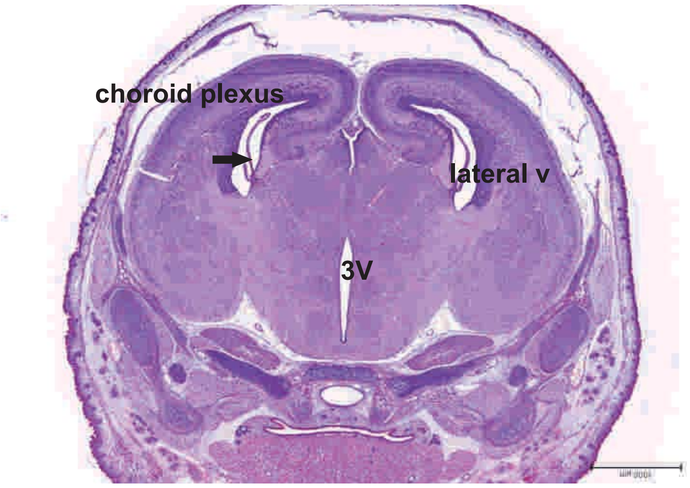

#4 Midbrain x2 29732

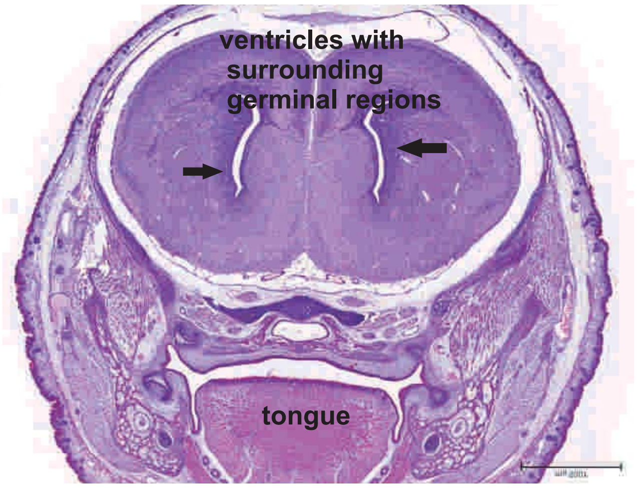

#4 Forebrain x2 29726

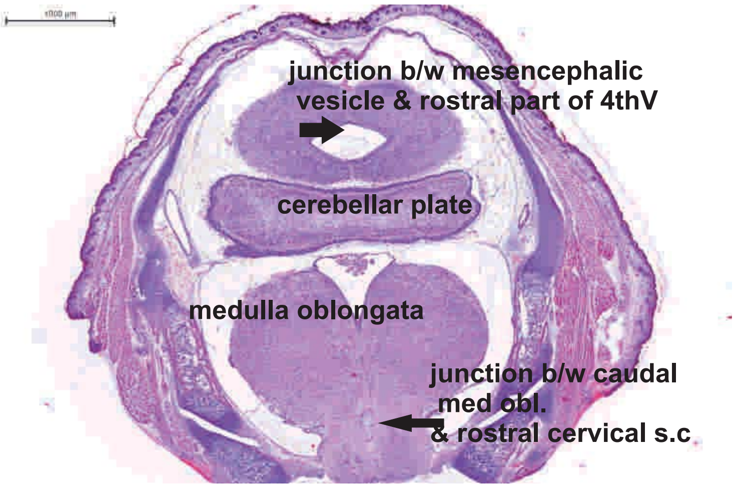

#4 hindbrain x2 29747

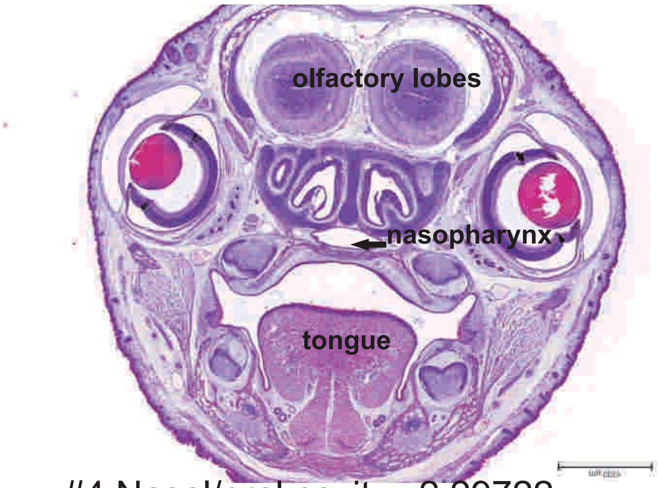

#4 Nasal/oral cavity x2 29722

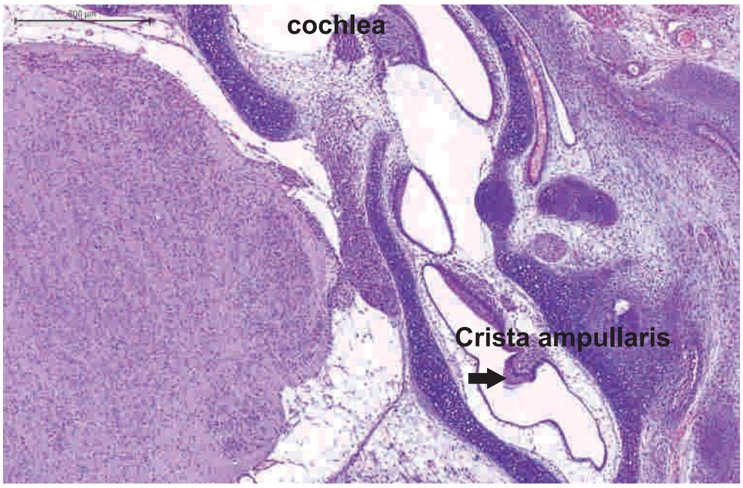

#4 Inner ear x10 29740
