# Supplementary figures and images for: Contribution of the Two Genes Encoding Histone Variant H3.3 to Viability and Fertility in Mice
Source: PLoS Genet. 2015 Feb 12;11(2):e1004964. doi: 10.1371/journal.pgen.1004964 (PMC4335506; doi:10.1371/journal.pgen.1004964)

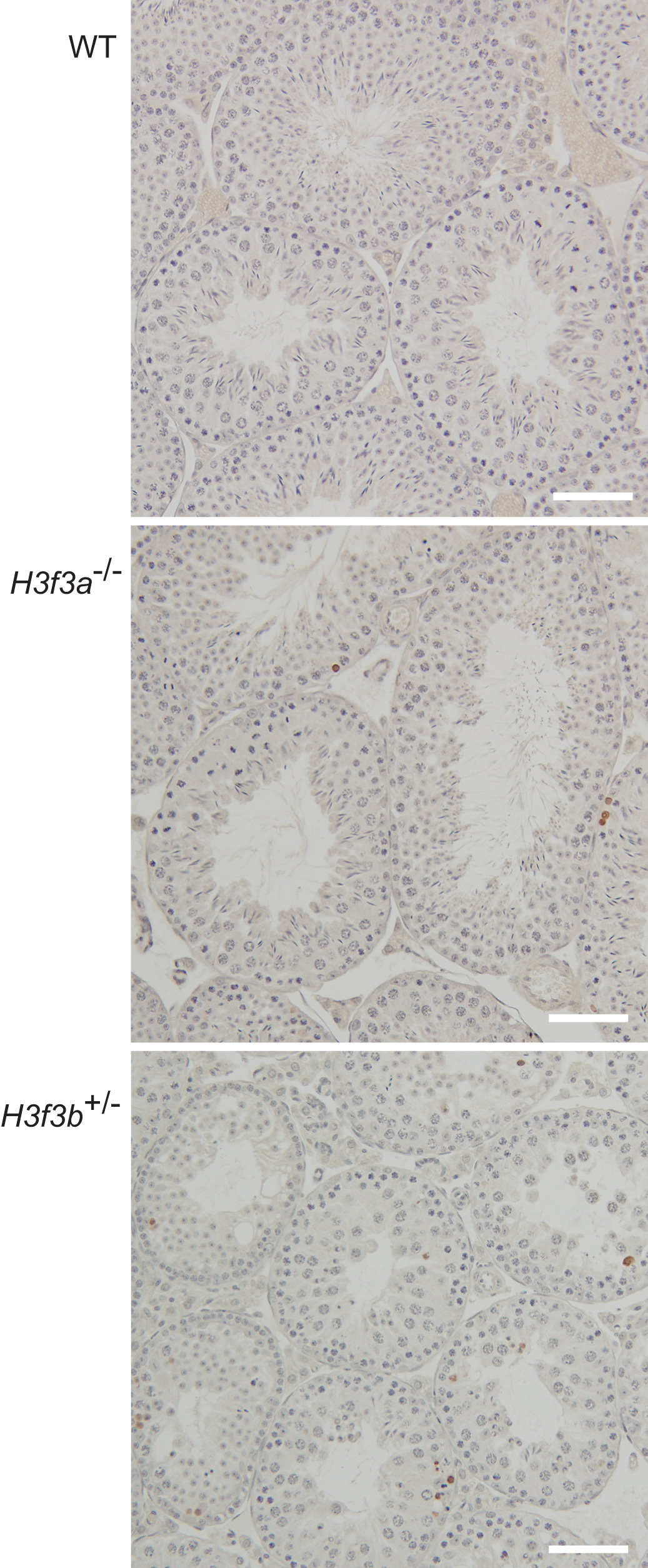

Supplement: S2 Fig — Bars, 25 μm. (TIF) [file pgen.1004964.s002.tif]

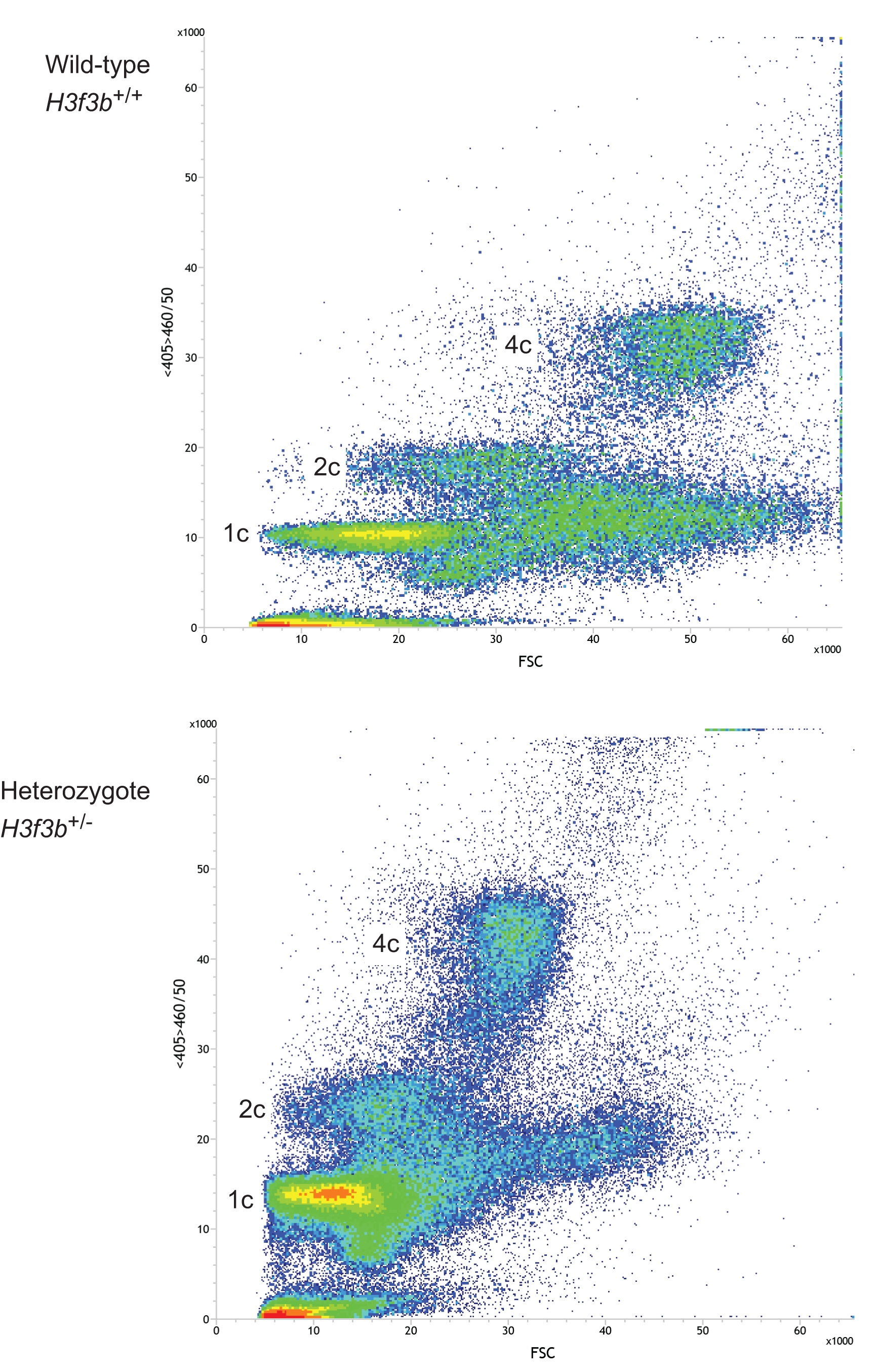

Supplement: S3 Fig — Propidium iodide-negative or live cells were plotted against Hoechst 33342 fluorescence (y-axis) versus forward scatter (x-axis) signals. Sorted larger 4c cells (pachytene spermatocytes) and smaller 1c cells (round spermatids) are indicated. (TIF) [file pgen.1004964.s003.tif]
